# The impact of different intensities of physical activity on aging: A Mendelian randomization study

**DOI:** 10.1097/MD.0000000000048055

**Published:** 2026-04-03

**Authors:** Hongxin Li, Wang Guo, Qiwen Nie, Qiang Tang, Hongyu Li

**Affiliations:** a Department of Rehabilitation Medicine, Heilongjiang University of Chinese Medicine, Harbin, Heilongjiang, China; b Department of Neurological Rehabilitation, The Second Affiliated Hospital of Heilongjiang University of Chinese Medicine, Harbin, Heilongjiang, China.

**Keywords:** aging, DNA methylation, exercise, Mendelian randomization analysis, telomere

## Abstract

The extant corpus of observational studies has yielded an inadequate body of evidence to establish a causal relationship between physical activity (PA) and the process of aging. This study aimed to determine the causal effects of different intensities of PA on biological aging using a 2-sample Mendelian randomization (MR) approach. Pooled data on PA and aging proxies were extracted from genome-wide association studies on individuals of European ancestry in order to perform MR analysis. Five MR analysis techniques were employed to reduce potential biases and ensure the robustness of the data, with the inverse-variance weighted method serving as the primary output. Sensitivity experiments were conducted to assess heterogeneity and pleiotropy in order to guarantee the results’ robustness. The results of the MR analysis demonstrated that genetically predicted walking activity was associated with longer telomere length (β = 0.118; 95% CI [0.022–0.215]; *P* = .01). Additionally, a significant negative causal relationship was identified between strenuous exercise and GrimAge (β = −1.432; 95% CI [−2.774 to −0.091]; *P* = .036). The robustness of these findings was further validated through conducting a series of sensitivity analyses. This research offers evidence supports a potential causal relationship between PA and slower aging. This connection is reflected in the preservation of telomere length and the slowing of GrimAge progression. As a result, encouraging PA could serve as an effective approach to mitigate the aging process.

## 1. Introduction

The global rise in population aging has intensified interest in elucidating the biological mechanisms underlying aging and in developing interventions to extend healthy lifespan. Ageing is a complex, multifactorial process involving genomic instability, telomere attrition, epigenetic alterations, loss of proteostasis, impaired macroautophagy, dysregulated nutrient sensing, mitochondrial dysfunction, cellular senescence, stem cell exhaustion, altered intercellular communication, chronic inflammation, and dysbiosis, among other hallmarks.^[1,2]^ These molecular and cellular changes are paralleled by shifts in aging biomarkers and an increased risk of age-related diseases, including cardiovascular, neurodegenerative, and autoimmune disorders.^[3]^ The prevalence of such chronic conditions continues to rise in older populations, often resulting in multimorbidity and substantially impacting both quality of life and life expectancy.^[4,5]^ Traditional measures such as chronological age are insufficient for accurately capturing the heterogeneity of the aging process. There is thus a growing need for alternative biomarkers that can more precisely reflect biological age and identify individuals at risk of accelerated aging.^[6]^ One promising approach is the assessment of epigenetic modifications, particularly DNA methylation-based “epigenetic clocks.”^[7]^ Epigenetic age acceleration, defined as the discrepancy between chronological age and DNA methylation-predicted age, has been shown to correlate with longevity,^[8]^ various pathological states, and disease risk factors.^[9–11]^ DNA methylation, involving the covalent addition of methyl groups to cytosine residues to form 5-methylcytosine, exhibits dynamic, age-related changes, with certain CpG sites showing a linear relationship with age, making them robust biomarkers of aging.^[12,13]^ At the cellular level, telomere shortening is a well-established marker of aging and cellular senescence.^[14]^ Frailty, a clinical syndrome frequently associated with hospitalization, mortality, and disability in older adults, is commonly quantified using the frailty index (FI), which reflects the cumulative burden of health deficits and provides a comprehensive measure of biological aging.^[15–17]^ The identification and validation of such biomarkers are critical for early detection and management of accelerated aging, as well as for the development of interventions aimed at prolonging healthy lifespan and improving quality of life. Physical activity (PA) has been shown to attenuate the accumulation of stochastic epigenetic mutations – termed the epigenetic mutation burden – during aging, particularly in women.^[18]^ Moreover, PA is associated with improved functional status and survival in older adults.^[19]^ Numerous studies have reported that leisure-time PA of varying intensities is inversely associated with accelerated aging, whereas occupational activity patterns may be positively associated with aging acceleration.^[20,21]^ Additionally, greater frequency and duration of PA are linked to slower biological aging.^[22]^ Conversely, some evidence suggests that sedentary behavior or walking may not be associated with biological age.^[23]^ However, the observational nature of most studies leaves them vulnerable to confounding and reverse causation, limiting their ability to establish definitive causal relationships and contributing to inconsistent findings.

Mendelian randomization (MR) leverages genetic variants as instrumental variables (IVs) to infer causal relationships between exposures and outcomes.^[24]^ Because genetic variants are randomly allocated at conception, MR analyses are less susceptible to confounding and reverse causation, providing more robust causal inference than traditional observational studies.^[25]^ Therefore, this study aimed to investigate the causal relationships between different intensities of PA and multiple biological aging indicators using MR analysis.

## 2. Methods

### 2.1. Research design

We systematically evaluated the causal relationship between PA and aging-related proxy indicators using a 2-sample MR framework. Genome-wide association study (GWAS) summary statistics were leveraged as IVs for both exposures and outcomes. Multiple sensitivity analyses were conducted to ensure the robustness and consistency of the findings. The effect of PA (exposure) on aging proxy indicators (outcomes) was assessed (data sources are detailed in Table 1). As this study utilized publicly available, summary-level GWAS data, no additional ethical approval or informed consent was required. The MR analysis was conducted in accordance with the STROBE-MR reporting guidelines.^[26]^

**Table 1:** Data sources for PA and aging proxy indicators.

Trait name		Consortium	Population	Sample size	GWAS ID
Exposure	Number of days/week walked 10 + min	MRC-IEU	European	454,783	ukb-b-4886
	Number of days/week of moderate physical activity 10 + min	MRC-IEU	European	440,266	ukb-b-4710
	Number of days/week of vigorous physical activity 10 + min	MRC-IEU	European	440,512	ukb-b-151
Outcome	DNA methylation GrimAge acceleration	NA	European	34,467	ebi-a-GCST90014288
	DNA methylation Hannum age acceleration	NA	European	34,449	ebi-a-GCST90014289
	DNA methylation PhenoAge acceleration	NA	European	34,463	ebi-a-GCST90014292
	Intrinsic epigenetic age acceleration	NA	European	34,461	ebi-a-GCST90014290
	Telomere length	NA	European	472,174	ieu-b-4879
	Frailty index	NA	European	175,226	ebi-a-GCST90020053

GWAS = genome-wide association study, PA = physical activity.

### 2.2. Data sources

PA data were derived from the UK Biobank, a large-scale, population-based prospective cohort designed to investigate genetic and nongenetic determinants of disease.^[27]^ PA was assessed via a touchscreen questionnaire, adapted from the International PA Questionnaire, administered to approximately 400,000 participants.^[28,29]^ Three categories of self-reported PA – light (LPA), moderate (MPA), and vigorous (VPA) – were evaluated. A total of 454,783 participants were included at LPA level after being specifically asked about their involvement in light PA using the following question: “In a typical week, how many days did you engage in walking for at least 10 minutes at a time, including walking at work, during commutes, and for sports or leisure activities?” 440,266 people were included in the MPA level, which questioned, “How many days in a typical week did you engage in moderate PA for 10 minutes or more, such as carrying light loads or cycling at a normal pace (excluding walking)?” “In a typical week, how many days did you engage in vigorous PA for 10 minutes or more, such as activities that induce sweating or heavy breathing, like fast cycling, aerobics, or heavy lifting?” was the question asked of participants in the VPA level, which included 440,512 participants. One noteworthy aspect of the study is the inclusion of walking as a sample of low-intensity physical exercise. Given that walking is a common low-intensity recreational activity, this addition could improve the questionnaire’s applicability.^[30]^

Epigenetic age data were obtained from a meta-analysis of 28 cohorts comprising 34,710 individuals of European ancestry (mean age 54.8 years, range 27.2–79.1; 57.3% female). Epigenetic age was estimated using Horvath Epigenetic Age Calculator or scripts provided by Steve Horvath and Ake Lu, identifying 137 DNA methylation loci associated with aging, including GrimAge, PhenoAge, HannumAge, and Intrinsic Epigenetic Age Acceleration.^[31–34]^

Telomere length (TL) GWAS summary statistics were derived from a meta-analysis of 472,174 individuals of European ancestry.^[35]^ TL was measured by quantitative PCR in UK Biobank participants, providing insight into the genetic determinants of aging.

FI GWAS summary data were obtained from the GWAS Catalog, including 175,226 individuals of European descent,^[36]^ comprising 10,616 Swedish TwinGene participants (aged 41–87 years) and 164,610 UK Biobank participants (aged 60–70 years).^[37]^

### 2.3. Screening tool variables

To assess causal associations between PA and aging proxy indicators, independent single nucleotide polymorphisms (SNPs) strongly associated with each exposure were selected as IVs. Instrument selection adhered to the core MR assumptions: IVs are robustly associated with the exposure; IVs are not associated with confounders of the exposure-outcome relationship; and IVs influence the outcome solely through the exposure. SNPs were initially selected at genome-wide significance (*P* <5 × 10^−8^). SNPs in linkage disequilibrium (distance >10,000 kb, *r*^2^ <0.001) were excluded. The remaining SNPs were harmonized across GWAS datasets for subsequent analyses.

Potential violations of MR assumptions were assessed using the LDTrait (https://ldlink.nih.gov/ldtrait), allowing exclusion of SNPs associated with known confounders or direct associations with outcomes. SNPs with ambiguous strand orientation (palindromic SNPs) or low minor allele frequency were also excluded. Outlier detection and correction were performed using radial MR and MR-PRESSO methods.

**Figure 1.**
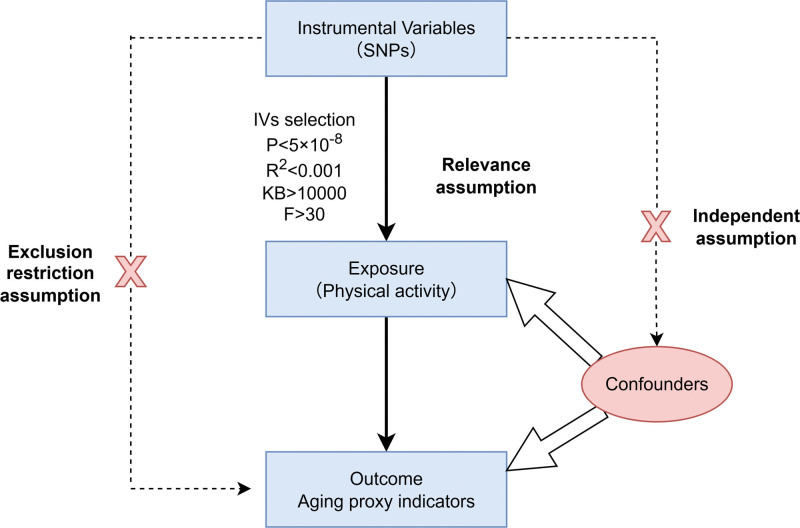
Study flow chart of the MR analysis. MR = Mendelian randomization.

Instrument strength was evaluated by calculating the F statistic for each SNP (*F* = β^2^/SE^2^, where β is the SNP-exposure effect size and SE is its standard error).^[38]^ An *F*-statistic >30 was considered indicative of sufficient instrument strength, minimizing the risk of weak instrument bias (see Fig. 1).

### 2.4. Statistical analyses

The primary method for evaluating the causal associations between genetically predicted PA intensities and 5 aging-related proxies in the 2-sample MR framework was the inverse-variance weighted (IVW) approach. To enhance the robustness and reliability of the findings, 4 additional MR methods – MR-Egger regression, weighted median (WM), simple mode, and weighted mode – were employed as sensitivity analyses. Heterogeneity among IVs was assessed using Cochran *Q*-statistic in both IVW and MR-Egger models, with a *P*-value >.05 indicating no significant heterogeneity. Potential horizontal pleiotropy was evaluated using the MR-Egger intercept, where a *P*-value <.05 was considered indicative of pleiotropy. The MR-PRESSO method was also applied to detect and correct for horizontal pleiotropy by comparing observed and expected residual sums of squares. Furthermore, leave-one-out analyses were conducted to determine whether any single SNP disproportionately influenced the results. All analyses were performed using R software (version 4.3.1; R Foundation for Statistical Computing, Vienna, Austria) with the TwoSampleMR package. Statistical significance was defined as a false discovery rate adjusted *P*-value <.05. As all data utilized were publicly available summary statistics, no additional ethical approval was required.

## 3. Result

### 3.1. Main results of Mendelian randomization

**Figure 2.**
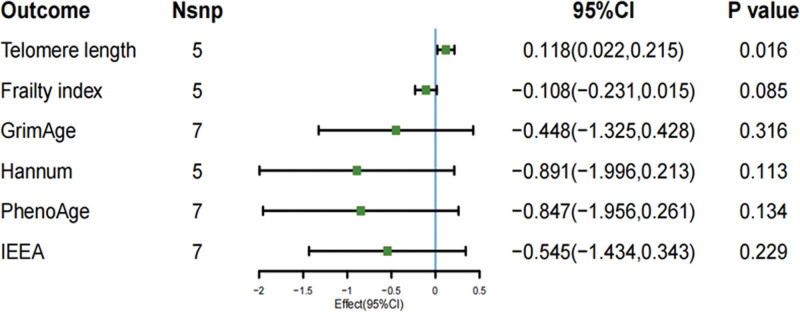
MR study of walk-on aging proxy indicators’ IVW result. IVW = inverse-variance weighted, MR = Mendelian randomization.

**Figure 3.**
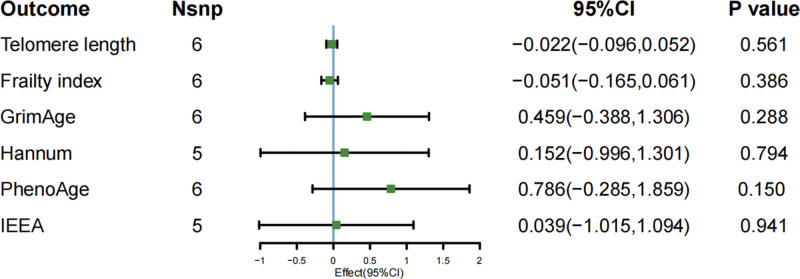
MR study of moderate PA on aging proxy indicators’ IVW result. IVW = inverse-variance weighted, MR = Mendelian randomization, PA = physical activity.

**Figure 4.**
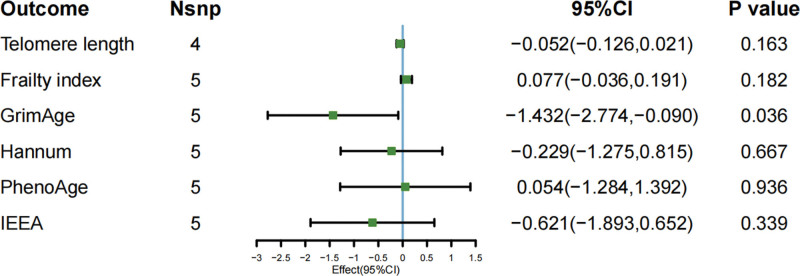
MR study of vigorous PA on aging proxy indicators’ IVW result. IVW = inverse-variance weighted, MR = Mendelian randomization, PA = physical activity.

The main results of IVW-based MR analysis showed that light PA and TL were causally related to light PA (β = 0.118; 95% CI [0.022–0.215]; *P* = .016). The results using the MR-Egger approach, however, were different but still pointed in the same direction (MR-Egger: β = 0.226; 95% CI [−1.013 to 1.465]; *P* = .744). Light PA did not show evidence of a causal association with FI (β = 0.077; 95% CI [−0.036 to 0.191]; *P* = .182), GrimAge (β = −0.448; 95% CI [−1.325 to 0.428]; *P* = .316), Hannum (β = −0.891; 95% CI [−1.996 to 0.213]; *P* = .113), PhenoAge (β = −0.847; 95% CI [−1.956 to 0.261]; *P* = .134), or IEEA (β = −0.545; 95% CI [−1.434 to 0.343]; *P* = .229), as illustrated in Figure 2. Regarding moderate intensity PA, no association was found with each of the aging proxy indicators TL (β = −0.022; 95 % CI [−0.096 to 0.052]; *P* = .561), FI (β = −0.051; 95 % CI [−0.165 to 0.061]; *P* = .368), GrimAge (β = 0.459; 95 % CI [−0.388 to 1.306]; *P* = .288),Hannum (β = 0.152; 95 % CI [−0.996 to 1.301]; *P* = .794), PhenoAge (β = 0.786; 95 % CI [−0.285 to 1.859]; *P* = .15), IEEA (β = 0.039; 95 % CI [−1.015 to 1.094]; *P* = .941) were causally related to each other, as demonstrated in Figure 3. A causal relationship was identified between VPA and GrimAge (β = −1.432; 95% CI [−2.774 to −0.091]; *P* = .036), a finding that was further supported by the WM method (β = −1.806; 95% CI [−3.268 to −0.343]; *P* = .015). However, the MR-Egger analysis produced similar results that were not statistically significant (β = −2.669; 95% CI [−12.929 to 7.589]; *P* = .645). Beyond GrimAge, no other aging-related indicators showed evidence of a causal link with VPA: TL (β = −0.052; 95% CI [−0.126 to 0.021]; *P* = .163), FI (β = 0.077; 95% CI [−0.036 to 0.191]; *P* = .182), Hannum (β = −0.229; 95% CI [−1.275 to 0.815]; *P* = .666), PhenoAge (β = 0.054; 95% CI [−1.284 to 1.392]; *P* = .936), and IEEA (β = −0.621; 95% CI [−1.893 to 0.652]; *P* = .339), as demonstrated in Figure 4. The correlation between SNPs related to PA and aging may be demonstrated through the implementation of forest plots and scatter plots, which offer a visual representation of the relationship between these variables. This analysis is further elaborated in Appendix S1, Supplemental Digital Content, https://links.lww.com/MD/R586.

### 3.2. Sensitivity analysis

In order to analyze horizontal pleiotropy, MR-egger analysis of intercepts was used, *P* >.05 indicating no horizontal pleiotropy. In order to further test the possibility of horizontal pleiotropy, MR-PRESSO was used, which involves the exclusion of outliers from the data. As confirmed by funnel plot analysis (Appendix S1, Supplemental Digital Content, https://links.lww.com/MD/R586), there was no observable horizontal pleiotropy for any of the outcomes.^[39]^ The results of the sensitivity analyses, did not show any evidence of the presence of heterogeneity according to Cochrane *Q*-test (*P* >.05), as shown in Table 2. Furthermore, the *F*-statistic for each IVs was >30, indicating that there was no weak instrumental bias in this MR study. The leave-one-out sensitivity analysis plots showed that there were no individual SNPs that could have influenced causality, thus reinforcing the robustness of our conclusions (Appendix S1, Supplemental Digital Content, https://links.lww.com/MD/R586).

**Table 2:** Sensitivity analyses for the causal associations of PA with aging proxy indicators.

Exposures	Outcomes	Cochran *Q (P*)	MR-egger (*P*)	MR-PRESSO (*P*)
Walk	Telomere length	.099	.875	.167
	Frailty index	.332	.339	.400
	GrimAge	.986	.493	.987
	Hannum	.342	.299	.373
	PhenoAge	.668	.757	.690
	IEEA	.624	.721	.602
Moderate physical activity	Telomere length	.100	.867	.125
	Frailty index	.179	.367	.196
	GrimAge	.660	.953	.670
	Hannum	.193	.133	.228
	PhenoAge	.833	.804	.842
	IEEA	.181	.856	.191
Vigorous physical activity	Telomere length	.920	.982	.913
	Frailty index	.443	.977	.476
	GrimAge	.175	.826	.261
	Hannum	.910	.868	.901
	PhenoAge	.791	.735	.806
	IEEA	.230	.759	.317

MR-PRESSO = Mendelian randomization-pleiotropy residual sum and outlier, PA = physical activity.

## 4. Discussion

Recent studies have leveraged GWAS data and MR approaches to investigate the causal relationships between different intensities of PA and various biological aging markers in European cohorts. For instance, Dempsey et al and Chen et al applied MR methods in UK and Chinese populations, respectively, and identified potential causal associations between walking pace or walking activity and both TL and epigenetic age acceleration.^[40,41]^ Similarly, Zhao et al systematically evaluated the relationships between leisure-time PA, sedentary behavior, and biological aging using both genetic correlation and MR analyses.^[42]^ Collectively, these studies provide robust genetic evidence supporting the impact of PA on the aging process. This study provides several novel contributions. First, unlike previous MR studies focusing on a single PA phenotype, we systematically evaluated light, moderate, and vigorous activity simultaneously. Second, we incorporated multiple biological aging indicators, including TL, FI, and 4 epigenetic clocks, enabling a multidimensional assessment of aging. Third, we applied a comprehensive MR framework integrating multiple sensitivity methods to strengthen causal inference. These design features enhance both methodological rigor and biological interpretability.

Our results reveal distinct associations between PA and aging biomarkers. Two-sample MR analysis identified a potential causal link between low-intensity exercise and TL, suggesting that activities such as walking may promote longer telomeres. Although MR-Egger analysis yielded results in the same direction, statistical significance was not achieved, likely due to reduced precision from weaker IVs or unaccounted pleiotropy. The consistency across multiple MR methods supports the hypothesis that walking may positively influence TL, though caution is warranted in interpreting the robustness of these causal inferences.

No significant causal relationship was observed between moderate PA and any of the aging proxy indicators examined (TL, FI, GrimAge, Hannum, PhenoAge, or intrinsic epigenetic age acceleration) (all *P* >.05). This lack of association may reflect insufficient intensity or duration of moderate activity to elicit measurable effects on these aging markers, or the possibility that the relationship is mediated by factors not captured in this analysis. In contrast, vigorous PA was significantly associated with reduced GrimAge acceleration (β = −1.432; 95% CI: −2.774 to −0.091; *P* = .036), indicating that higher levels of vigorous activity may decelerate epigenetic aging as measured by GrimAge. However, the MR-Egger analysis, while showing a similar trend, did not reach statistical significance (β = −2.669; *P* = .645), underscoring the need for cautious interpretation due to potential pleiotropy and limited statistical power.

Methodologically, the IVW approach offers greater estimation accuracy and statistical power when core MR assumptions are met, but is more susceptible to horizontal pleiotropy.^[43]^ The MR-Egger method, by incorporating an intercept term, can detect and adjust for pleiotropy, though it is less statistically efficient and may lack power in the presence of weak instruments or small sample sizes.^[44,45]^ The WM approach provides more robust estimates even when up to half of the instruments are invalid, enhancing the reliability of causal inference.^[46]^

The biology of aging is characterized by telomere attrition,^[47]^ increased frailty,^[48]^ and accelerated epigenetic aging.^[49]^ Observational studies and clinical trials have reported inconsistent findings regarding the effects of PA on these markers.^[50]^ Our results are partially consistent with previous research, some of which have suggested that PA increases TL,^[51–54]^ while others have found no association.^[54–56]^ Such discrepancies may be attributable to limited sample sizes, biases in observational research, or differences in study design. Notably, most prior studies have examined the effect of a single exercise intensity at a specific time point, limiting their ability to capture the complex causal relationship between PA and TL. Consistent with findings from NHANES-based observational research, our study found no association between moderate or vigorous activity and TL, but did identify a causal link between low-intensity activity (walking) and TL.^[57]^ Conversely, another observational study reported that while all exercise intensities were associated with increased life expectancy, TL mediated this association only for moderate and vigorous activity.^[58]^ This discrepancy may be due to differences in the number of SNPs included in the analyses.

Importantly, we identified a causal relationship only between vigorous PA and the epigenetic clock GrimAge, which integrates DNA methylation-based markers of plasma proteins and smoking history, and has been shown to predict morbidity and mortality more accurately than other epigenetic clocks.^[32]^ Previous studies have demonstrated that higher-intensity PA is associated with deceleration of the epigenetic clock and reduced mortality.^[59–62]^ Vigorous activity has also been shown to be superior to moderate activity in improving cardiorespiratory and metabolic fitness,^[63–66]^ and is a more effective predictor of mortality and morbidity.^[67,68]^

Our MR analyses did not reveal a causal relationship between PA and FI, despite evidence from observational studies suggesting beneficial effects of PA on frailty. For example, a study of 1635 older adults found no significant difference in frailty risk between sedentary and moderate-intensity activity.^[69]^ Previous MR studies have also reported inconsistent results regarding the effect of PA on frailty, which may be due to differences in self-report measures or the strength of genetic instruments.^[70,71]^

Despite these findings, several limitations should be acknowledged. First, the lack of significant associations for most PA intensities highlights the complexity of aging biology and the potential for residual confounding. Second, discrepancies between IVW and MR-Egger results suggest possible bias due to horizontal pleiotropy, which cannot be fully excluded and warrants further investigation. Third, the categorization of PA into LPA, MPA, and VPA may not fully capture the diversity of individual activity patterns and their biological effects.

In summary, our findings highlight the selective effects of PA on aging proxy indicators, with low-intensity activity (walking) associated with longer telomeres and vigorous activity associated with reduced epigenetic aging (GrimAge). Future research should explore the underlying mechanisms, including oxidative stress regulation, inflammation, and metabolic adaptation. Longitudinal and interventional studies are needed to validate these causal relationships and assess their generalizability.

## 5. Conclusion

Modifiable lifestyle factors, such as PA, play a critical role in shaping the trajectory of biological aging. Aging is a complex, multifaceted process affecting multiple physiological systems and contributing to functional decline. Our MR analyses provide genetic evidence that PA can attenuate the aging process, with walking associated with reduced telomere attrition and vigorous exercise linked to slower progression of GrimAge. Collectively, these findings highlight the nuanced effects of PA on specific aging biomarkers and underscore its potential to inform personalized strategies for promoting healthy aging.

## Acknowledgments

We would like to thank the researchers and participants of all GWAS from which the summary statistics datasets were used in this study.

## Author contributions

**Conceptualization:** Hongxin Li, Hongyu Li.

**Data curation:** Hongxin Li, Wang Guo.

**Investigation:** Qiwen Nie.

**Methodology:** Hongxin Li.

**Project administration:** Qiang Tang.

**Supervision:** Qiang Tang, Hongyu Li.

**Validation:** Qiwen Nie.

**Writing – original draft:** Hongxin Li, Wang Guo.

**Writing – review & editing:** Qiang Tang, Hongyu Li.

## Supplementary Material
