# Supplementary material for: The impact of different intensities of physical activity on aging: A Mendelian randomization study
Source: Medicine (Baltimore). 2026 Apr 3;105(14):e48055. doi: 10.1097/MD.0000000000048055 (PMC13052978; doi:10.1097/MD.0000000000048055)
Supplement: Supplementary file 1 [file medi-105-e48055-s001.pdf]

Appendix S1. Mendelian randomization analysis of physical activity on aging proxy indicators (Scatter plots, forest plot, funnel plots, leave-one-out)

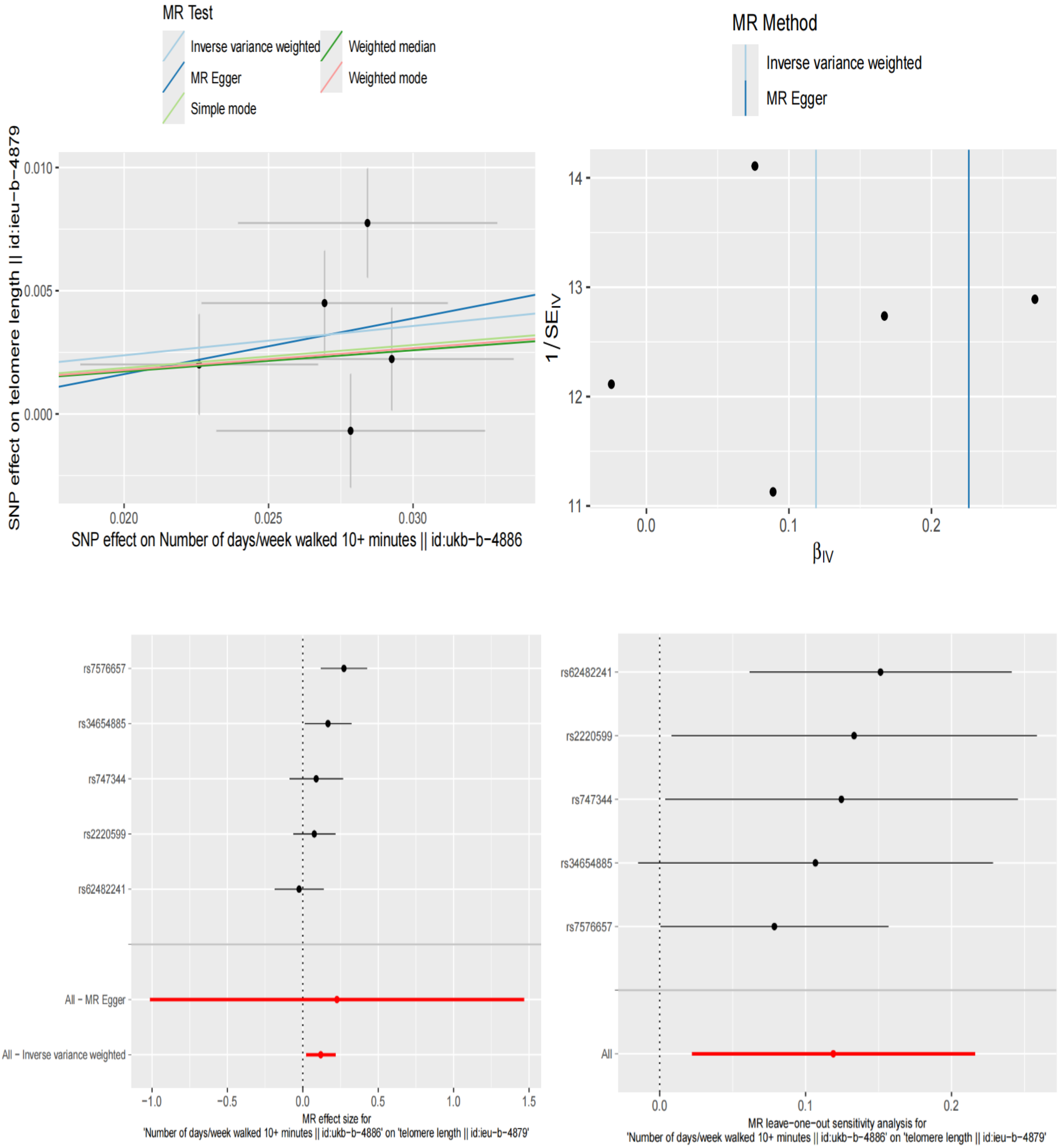
MR Test
Inverse variance weighted
MR Egger
Simple mode
Weighted median
Weighted mode
SNP effect on telomere length || id:ieu-b-4879
0.010
0.005
0.000
0.020
0.025
0.030
SNP effect on Number of days/week walked 10+ minutes || id:ukb-b-4886
MR Method
Inverse variance weighted
MR Egger
1 / SE$_{IV}$
14
13
12
11
0.0
0.1
0.2
β_{IV}
rs7576657
rs34654885
rs747344
rs2220599
rs62482241
All - MR Egger
All - Inverse variance weighted
-1.0
-0.5
0.0
0.5
1.0
1.5
MR effect size for
'Number of days/week walked 10+ minutes || id:ukb-b-4886' on 'telomere length || id:ieu-b-4879'
rs62482241
rs2220599
rs747344
rs34654885
rs7576657
All
0.0
0.1
0.2
MR leave-one-out sensitivity analysis for
'Number of days/week walked 10+ minutes || id:ukb-b-4886' on 'telomere length || id:ieu-b-4879'

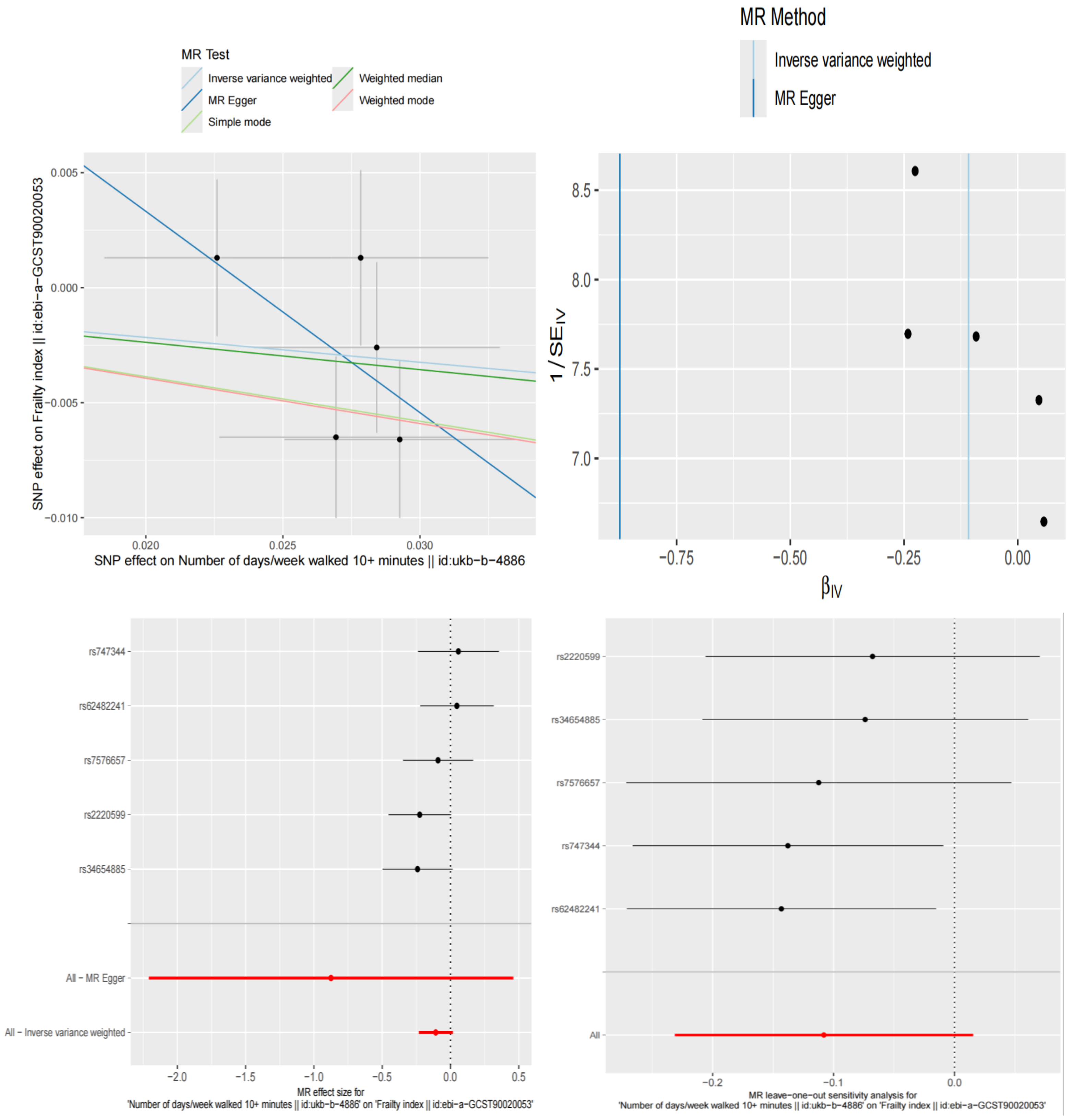
MR Test
Inverse variance weighted
Weighted median
MR Egger
Weighted mode
Simple mode
SNP effect on Frailty index || id:ebi−a−GCST90020053
0.005
0.000
−0.005
−0.010
0.020
0.025
0.030
SNP effect on Number of days/week walked 10+ minutes || id:ukb−b−4886
MR Method
Inverse variance weighted
MR Egger
1 / SE_IV
8.5
8.0
7.5
7.0
−0.75
−0.50
−0.25
0.00
β_IV
rs747344
rs62482241
rs7576657
rs2220599
rs34654885
All − MR Egger
All − Inverse variance weighted
−2.0
−1.5
−1.0
−0.5
0.0
0.5
MR effect size for
'Number of days/week walked 10+ minutes || id:ukb−b−4886' on 'Frailty index || id:ebi−a−GCST90020053'
rs2220599
rs34654885
rs7576657
rs747344
rs62482241
All
−0.2
−0.1
0.0
MR leave−one−out sensitivity analysis for
'Number of days/week walked 10+ minutes || id:ukb−b−4886' on 'Frailty index || id:ebi−a−GCST90020053'

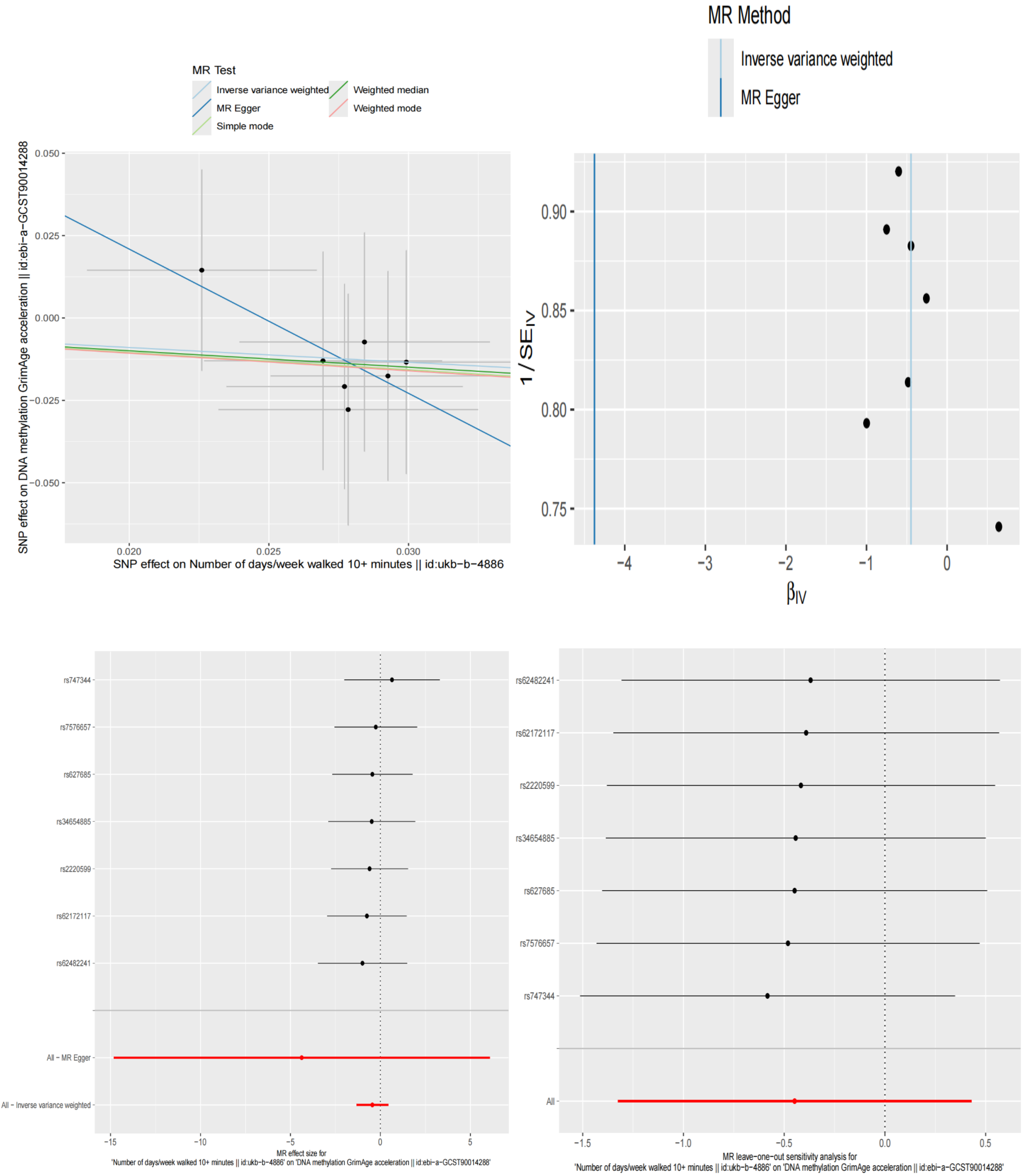
MR Test
Inverse variance weighted
MR Egger
Simple mode
Weighted median
Weighted mode
SNP effect on DNA methylation GrimAge acceleration || id:ebi-a-GCST90014288
SNP effect on Number of days/week walked 10+ minutes || id:ukb-b-4886
MR Method
Inverse variance weighted
MR Egger
1 / SE_IV
β_IV
rs747344
rs7576657
rs627685
rs34654885
rs2220599
rs62172117
rs62482241
All − MR Egger
All − Inverse variance weighted
MR effect size for
'Number of days/week walked 10+ minutes || id:ukb-b-4886' on 'DNA methylation GrimAge acceleration || id:ebi-a-GCST90014288'
All
MR leave-one-out sensitivity analysis for
'Number of days/week walked 10+ minutes || id:ukb-b-4886' on 'DNA methylation GrimAge acceleration || id:ebi-a-GCST90014288'

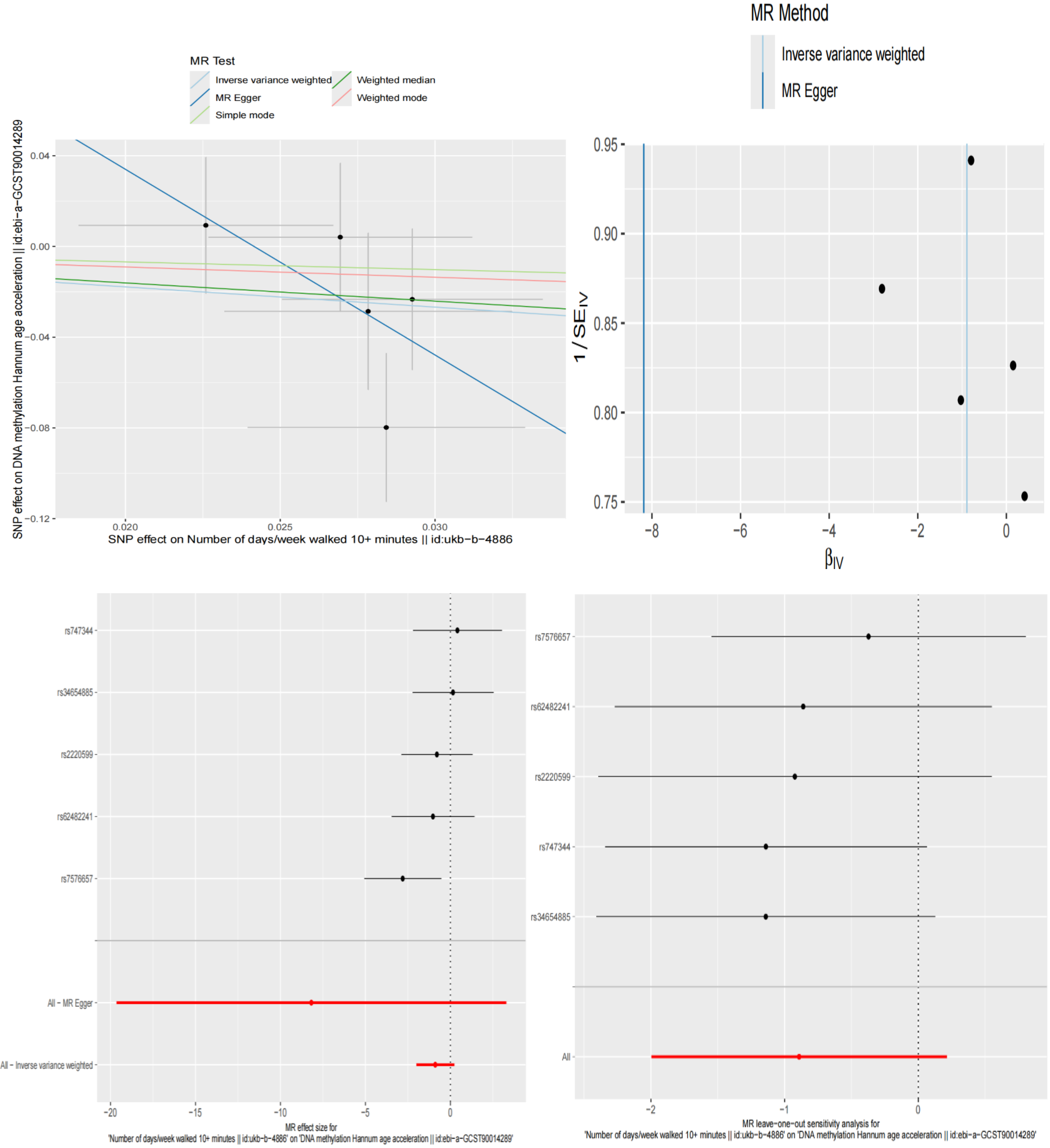

MR Test
Inverse variance weighted
MR Egger
Simple mode
Weighted median
Weighted mode
SNP effect on DNA methylation Hannum age acceleration || id:ebi-a-GCST90014289
SNP effect on Number of days/week walked 10+ minutes || id:ukb-b-4886
MR Method
Inverse variance weighted
MR Egger
1 / SE$_{IV}$
β$_{IV}$
rs747344
rs34654885
rs2220599
rs62482241
rs7576657
All − MR Egger
All − Inverse variance weighted
MR effect size for
'Number of days/week walked 10+ minutes || id:ukb-b-4886' on 'DNA methylation Hannum age acceleration || id:ebi-a-GCST90014289'
rs7576657
rs62482241
rs2220599
rs747344
rs34654885
All
MR leave-one-out sensitivity analysis for
'Number of days/week walked 10+ minutes || id:ukb-b-4886' on 'DNA methylation Hannum age acceleration || id:ebi-a-GCST90014289'

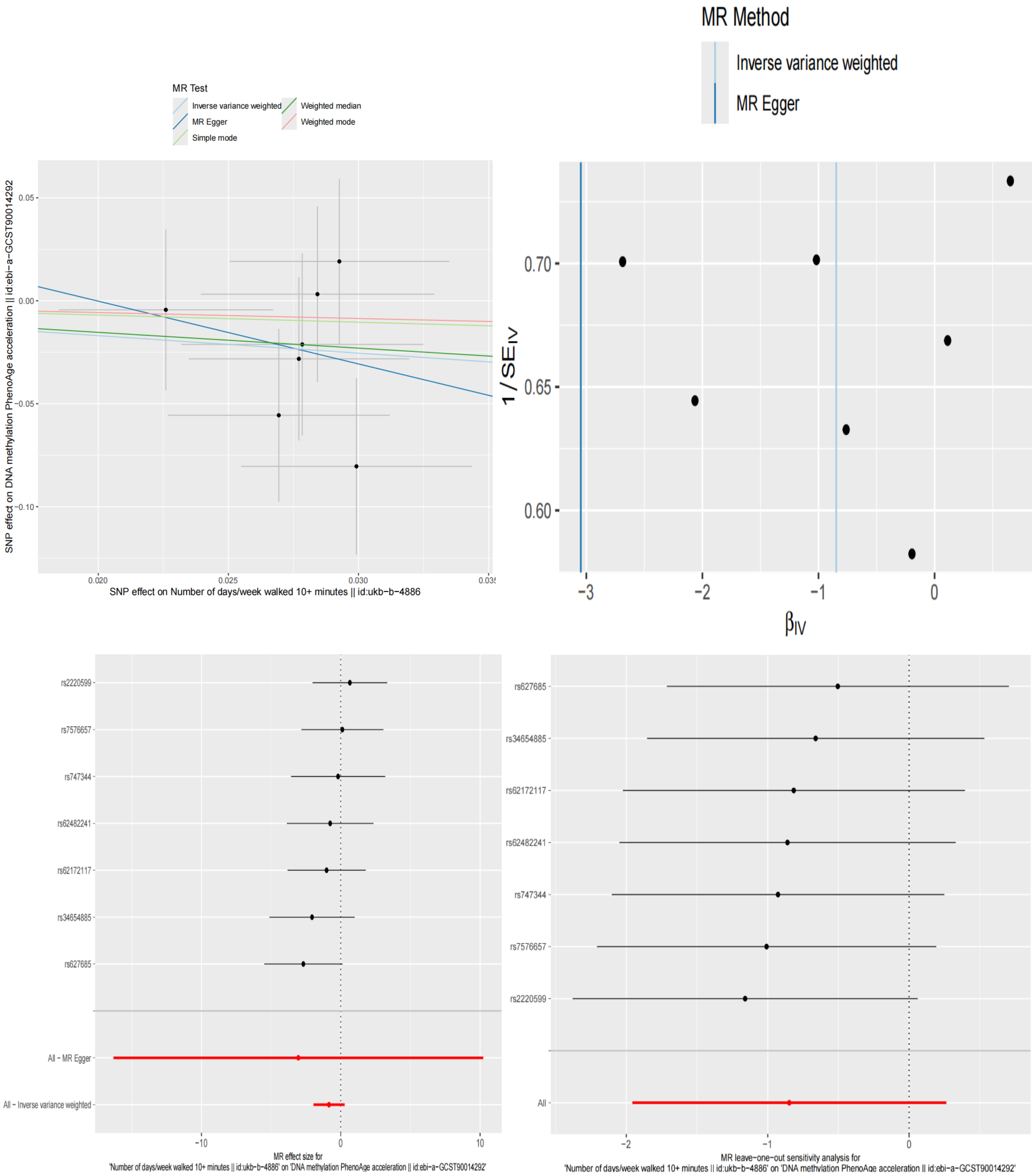
MR Test
Inverse variance weighted
MR Egger
Simple mode
Weighted median
Weighted mode
SNP effect on DNA methylation PhenoAge acceleration || id:ebi-a-GCST90014292
SNP effect on Number of days/week walked 10+ minutes || id:ukb-b-4886
MR Method
Inverse variance weighted
MR Egger
1 / SE$_{IV}$
β$_{IV}$
rs2220599
rs7576657
rs747344
rs62482241
rs62172117
rs34654885
rs627685
All - MR Egger
All - Inverse variance weighted
MR effect size for
'Number of days/week walked 10+ minutes || id:ukb-b-4886' on 'DNA methylation PhenoAge acceleration || id:ebi-a-GCST90014292'
rs627685
rs34654885
rs62172117
rs62482241
rs747344
rs7576657
rs2220599
All
MR leave-one-out sensitivity analysis for
'Number of days/week walked 10+ minutes || id:ukb-b-4886' on 'DNA methylation PhenoAge acceleration || id:ebi-a-GCST90014292'

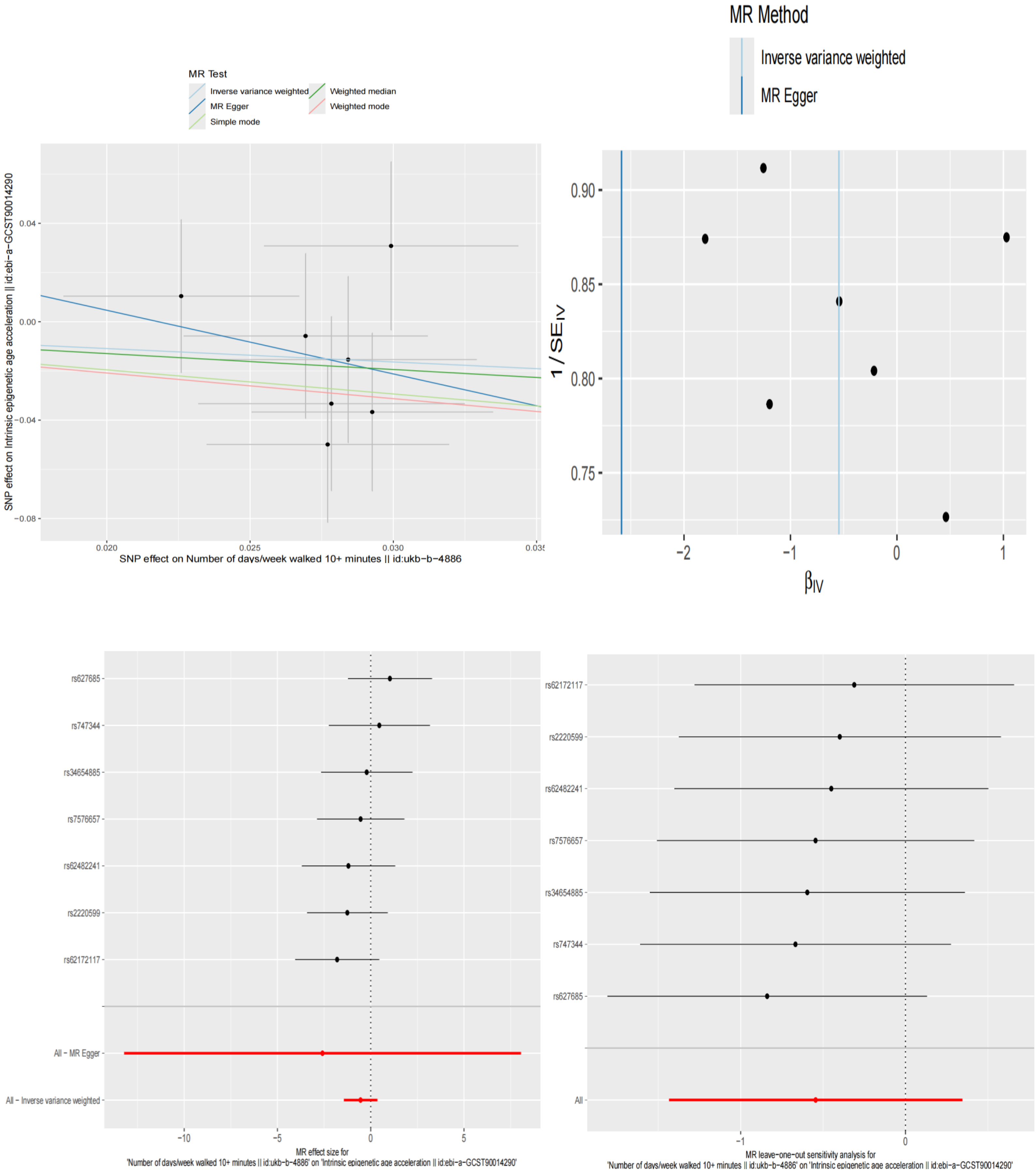

MR Test
Inverse variance weighted
MR Egger
Simple mode
Weighted median
Weighted mode
SNP effect on Intrinsic epigenetic age acceleration || id:ebi-a-GCST90014290
0.04
0.00
-0.04
-0.08
0.020
0.025
0.030
SNP effect on Number of days/week walked 10+ minutes || id:ukb-b-4886
MR Method
Inverse variance weighted
MR Egger
0.90
0.85
0.80
0.75
$1/SE_{IV}$
-2
-1
0
1
β_{IV}
rs627685
rs747344
rs34654885
rs7576657
rs62482241
rs2220599
rs62172117
All - MR Egger
All - Inverse variance weighted
-10
-5
0
5
MR effect size for
'Number of days/week walked 10+ minutes || id:ukb-b-4886' on 'Intrinsic epigenetic age acceleration || id:ebi-a-GCST90014290'
rs62172117
rs2220599
rs62482241
rs7576657
rs34654885
rs747344
rs627685
All
-1
0
MR leave-one-out sensitivity analysis for
'Number of days/week walked 10+ minutes || id:ukb-b-4886' on 'Intrinsic epigenetic age acceleration || id:ebi-a-GCST90014290'

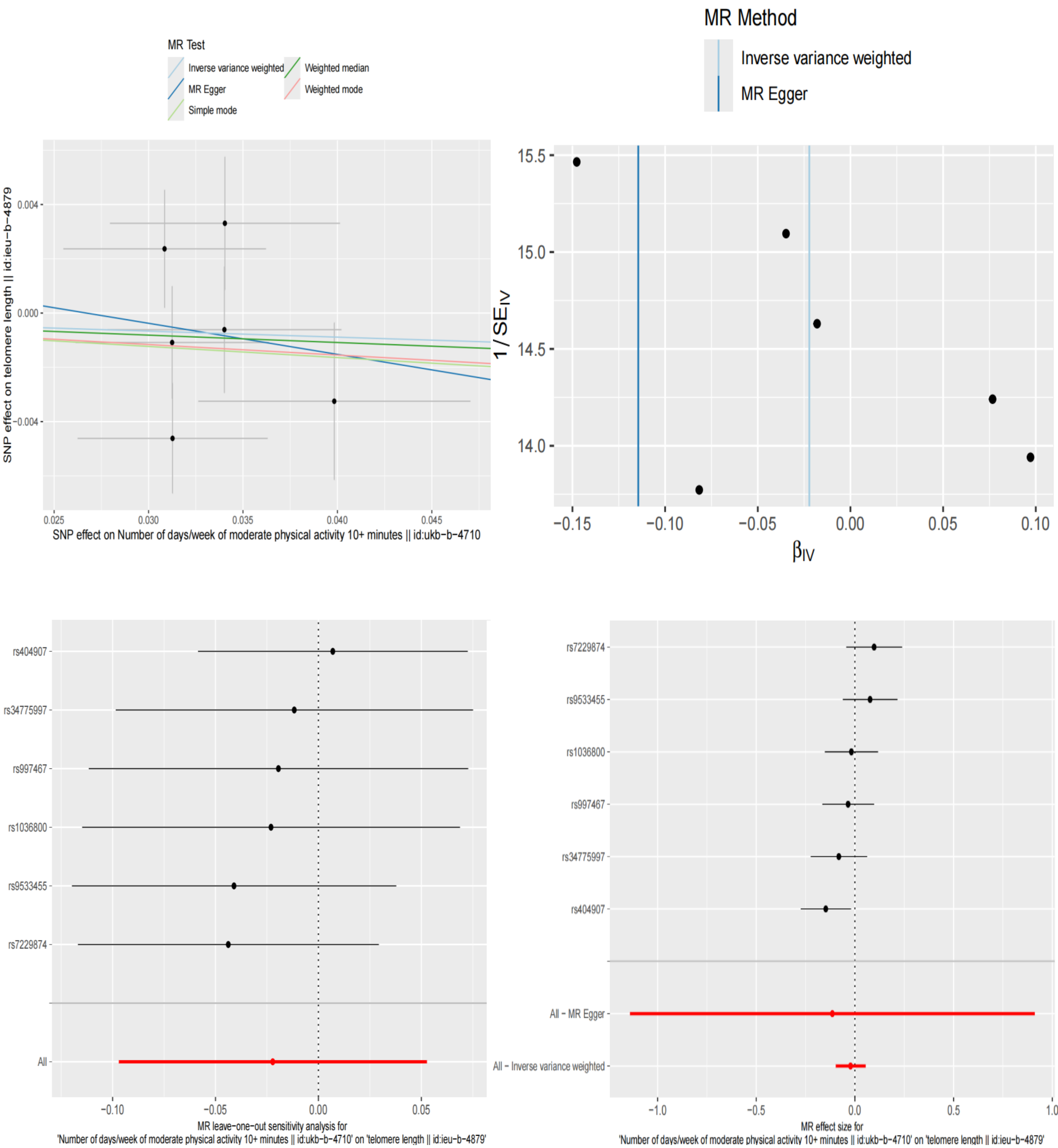
MR Test
Inverse variance weighted
Weighted median
MR Egger
Weighted mode
Simple mode
SNP effect on telomere length || id:ieu-b-4879
0.004
0.000
-0.004
0.025
0.030
0.035
0.040
0.045
SNP effect on Number of days/week of moderate physical activity 10+ minutes || id:ukb-b-4710
MR Method
Inverse variance weighted
MR Egger
15.5
15.0
14.5
14.0
1 / SE_IV
-0.15
-0.10
-0.05
0.00
0.05
0.10
β_IV
rs404907
rs34775997
rs997467
rs1036800
rs9533455
rs7229874
All
-0.10
-0.05
0.00
0.05
MR leave-one-out sensitivity analysis for
'Number of days/week of moderate physical activity 10+ minutes || id:ukb-b-4710' on 'telomere length || id:ieu-b-4879'
rs7229874
rs9533455
rs1036800
rs997467
rs34775997
rs404907
All - MR Egger
All - Inverse variance weighted
-1.0
-0.5
0.0
0.5
1.0
MR effect size for
'Number of days/week of moderate physical activity 10+ minutes || id:ukb-b-4710' on 'telomere length || id:ieu-b-4879'

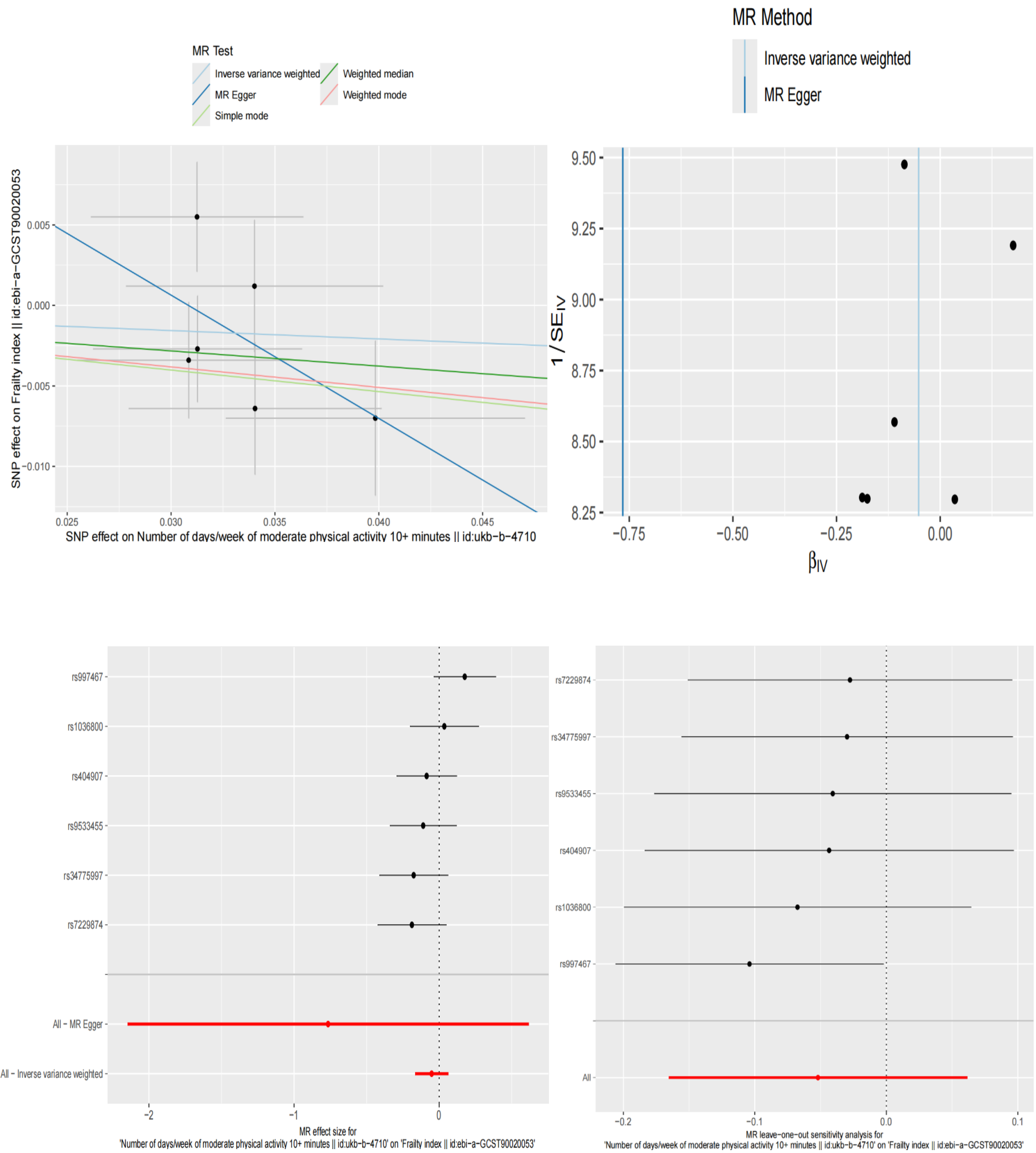
MR Test
Inverse variance weighted
MR Egger
Simple mode
Weighted median
Weighted mode
SNP effect on Frailty index || id:ebi-a-GCST90020053
0.005
0.000
−0.005
−0.010
0.025
0.030
0.035
0.040
0.045
SNP effect on Number of days/week of moderate physical activity 10+ minutes || id:ukb-b-4710
MR Method
Inverse variance weighted
MR Egger
1 / SE$_{IV}$
9.50
9.25
9.00
8.75
8.50
8.25
−0.75
−0.50
−0.25
0.00
β$_{IV}$
rs997467
rs1036800
rs404907
rs9533455
rs34775997
rs7229874
All − MR Egger
All − Inverse variance weighted
−2
−1
0
MR effect size for
'Number of days/week of moderate physical activity 10+ minutes || id:ukb-b-4710' on 'Frailty index || id:ebi-a-GCST90020053'
rs7229874
rs34775997
rs9533455
rs404907
rs1036800
rs997467
All
−0.2
−0.1
0.0
0.1
MR leave−one−out sensitivity analysis for
'Number of days/week of moderate physical activity 10+ minutes || id:ukb-b-4710' on 'Frailty index || id:ebi-a-GCST90020053'

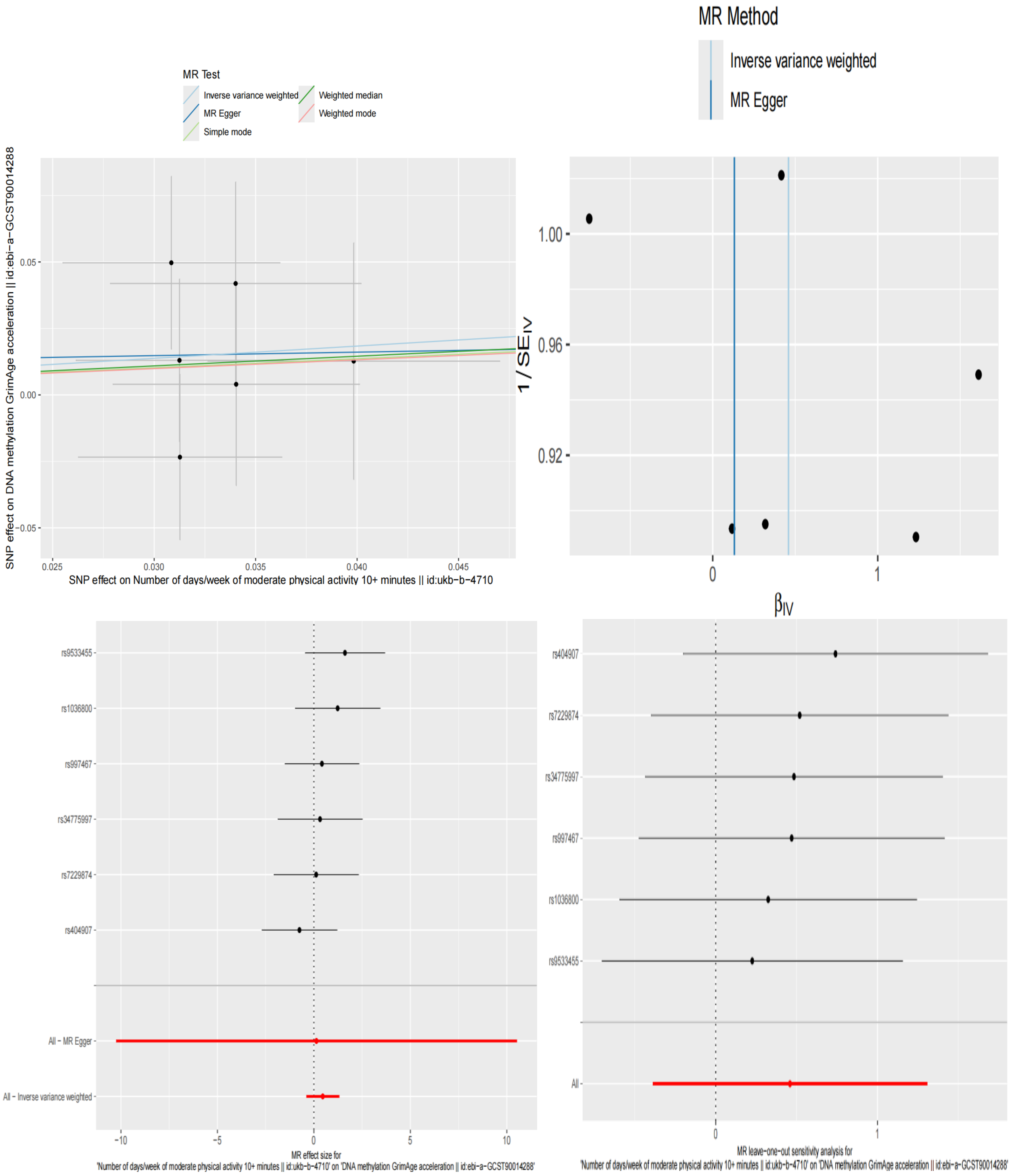
MR Test
Inverse variance weighted
Weighted median
MR Egger
Weighted mode
Simple mode
SNP effect on DNA methylation GrimAge acceleration || id:ebi-a-GCST90014288
SNP effect on Number of days/week of moderate physical activity 10+ minutes || id:ukb-b-4710
0.05
0.00
-0.05
0.025
0.030
0.035
0.040
0.045
MR Method
Inverse variance weighted
MR Egger
1 / SE$_{IV}$
1.00
0.96
0.92
0
1
β$_{IV}$
rs9533455
rs1036800
rs997467
rs34775997
rs7229874
rs404907
All - MR Egger
All - Inverse variance weighted
-10
-5
0
5
10
MR effect size for
'Number of days/week of moderate physical activity 10+ minutes || id:ukb-b-4710' on 'DNA methylation GrimAge acceleration || id:ebi-a-GCST90014288'
rs404907
rs7229874
rs34775997
rs997467
rs1036800
rs9533455
All
0
1
MR leave-one-out sensitivity analysis for
'Number of days/week of moderate physical activity 10+ minutes || id:ukb-b-4710' on 'DNA methylation GrimAge acceleration || id:ebi-a-GCST90014288'

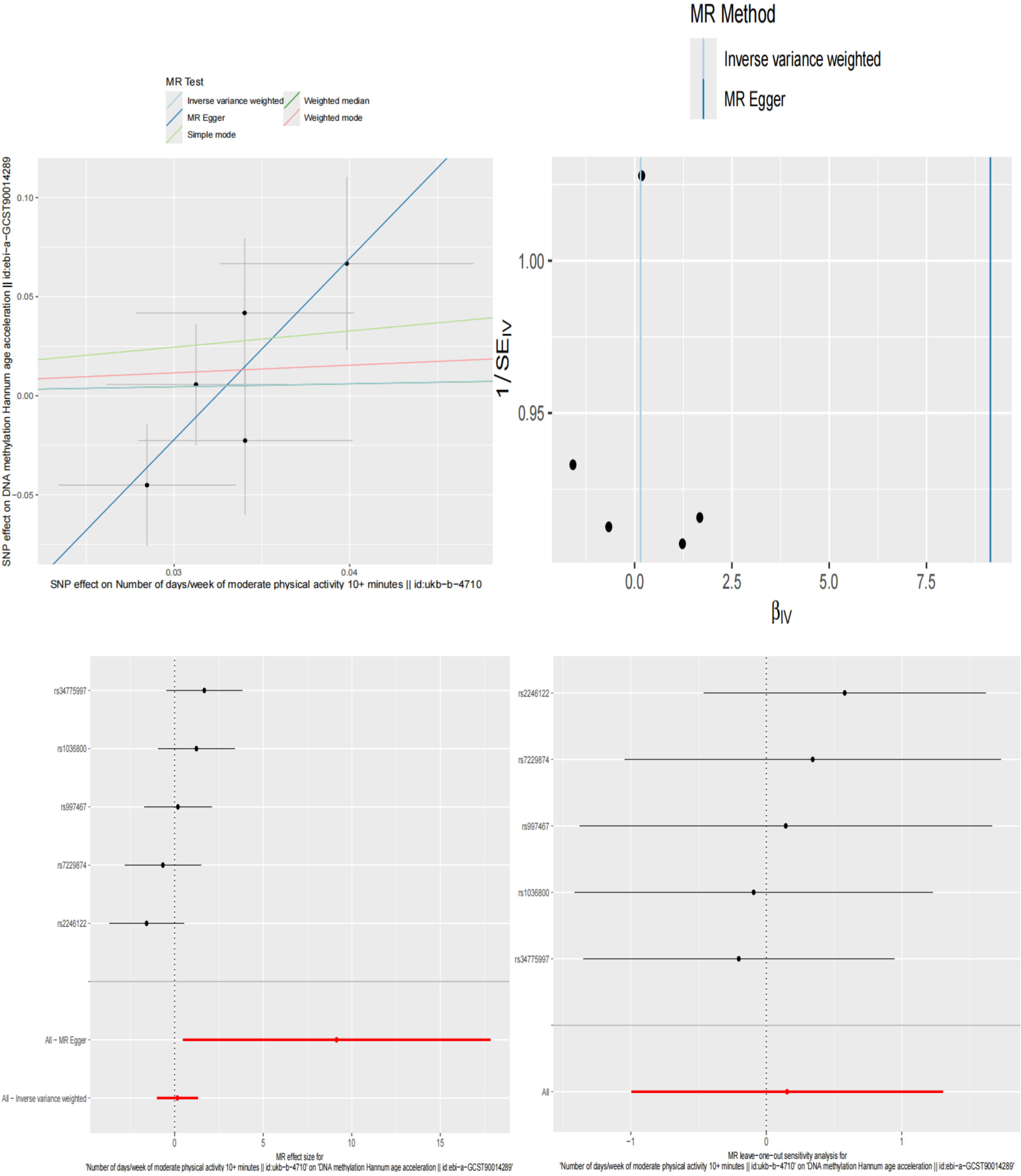
MR Test
Inverse variance weighted
MR Egger
Simple mode
Weighted median
Weighted mode
SNP effect on DNA methylation Hannum age acceleration || id:ebi-a-GCST90014289
SNP effect on Number of days/week of moderate physical activity 10+ minutes || id:ukb-b-4710
MR Method
Inverse variance weighted
MR Egger
1 / SE_IV
β_IV
rs34775997
rs1036800
rs997467
rs7229874
rs2246122
All - MR Egger
All - Inverse variance weighted
MR effect size for
'Number of days/week of moderate physical activity 10+ minutes || id:ukb-b-4710' on 'DNA methylation Hannum age acceleration || id:ebi-a-GCST90014289'
All
MR leave-one-out sensitivity analysis for
'Number of days/week of moderate physical activity 10+ minutes || id:ukb-b-4710' on 'DNA methylation Hannum age acceleration || id:ebi-a-GCST90014289'

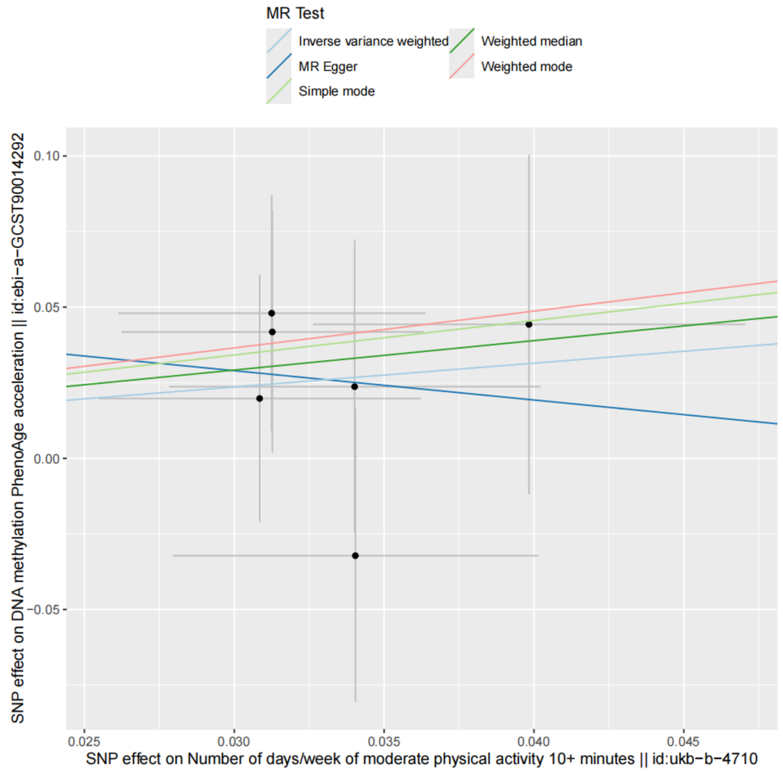
MR Test
Inverse variance weighted
MR Egger
Simple mode
Weighted median
Weighted mode
SNP effect on DNA methylation PhenoAge acceleration || id:ebi−a−GCST90014292
SNP effect on Number of days/week of moderate physical activity 10+ minutes || id:ukb−b−4710

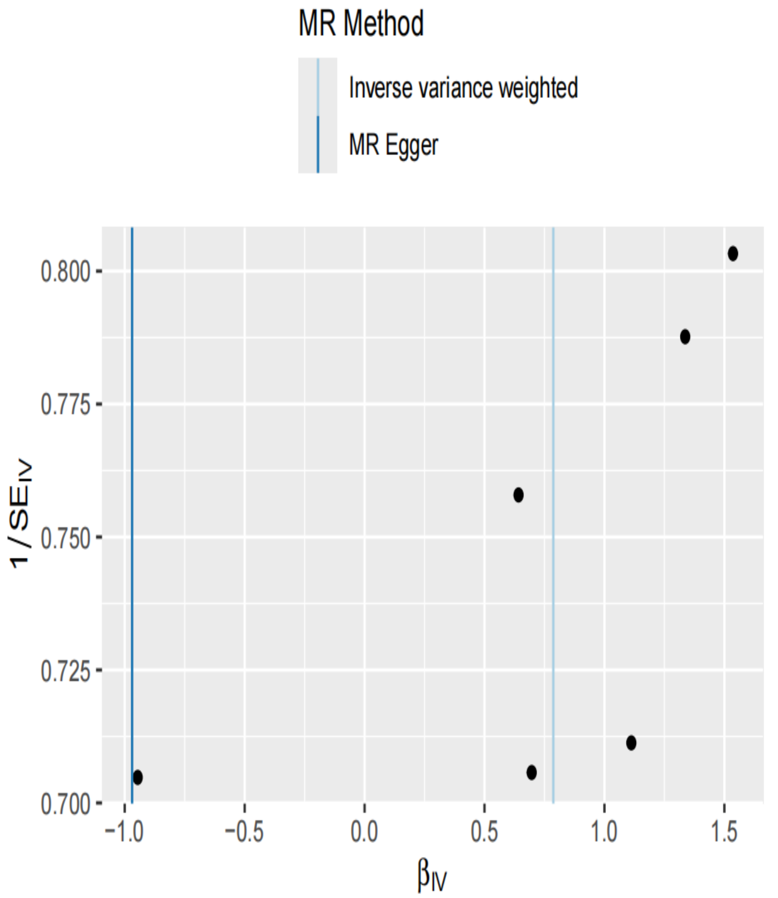
MR Method
Inverse variance weighted
MR Egger
1 / SE_IV
β_IV

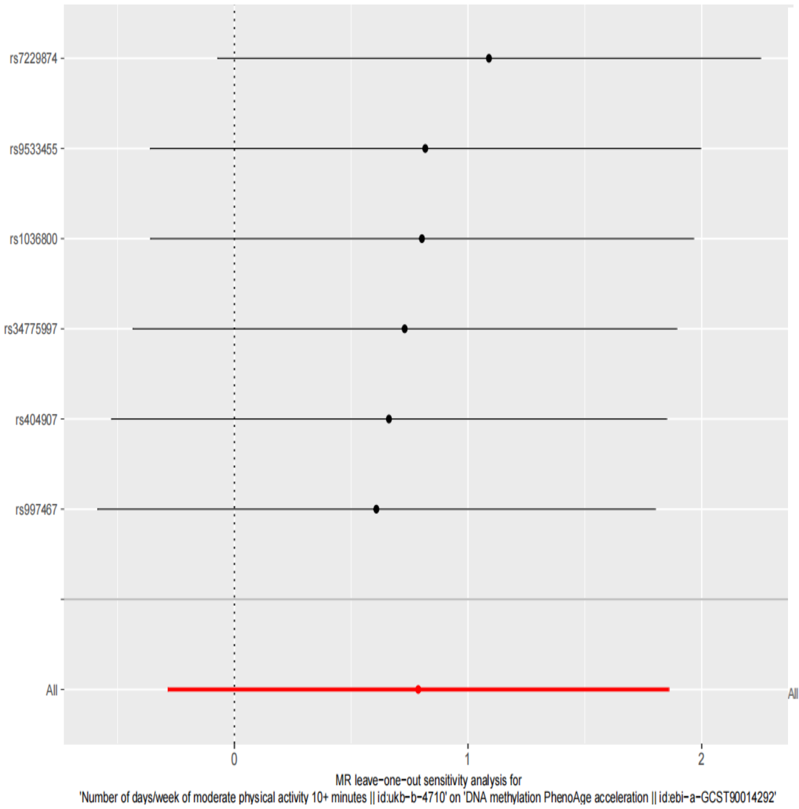
rs7229874
rs9533455
rs1036800
rs34775997
rs404907
rs997467
All
MR leave−one−out sensitivity analysis for
'Number of days/week of moderate physical activity 10+ minutes || id:ukb−b−4710' on 'DNA methylation PhenoAge acceleration || id:ebi−a−GCST90014292'

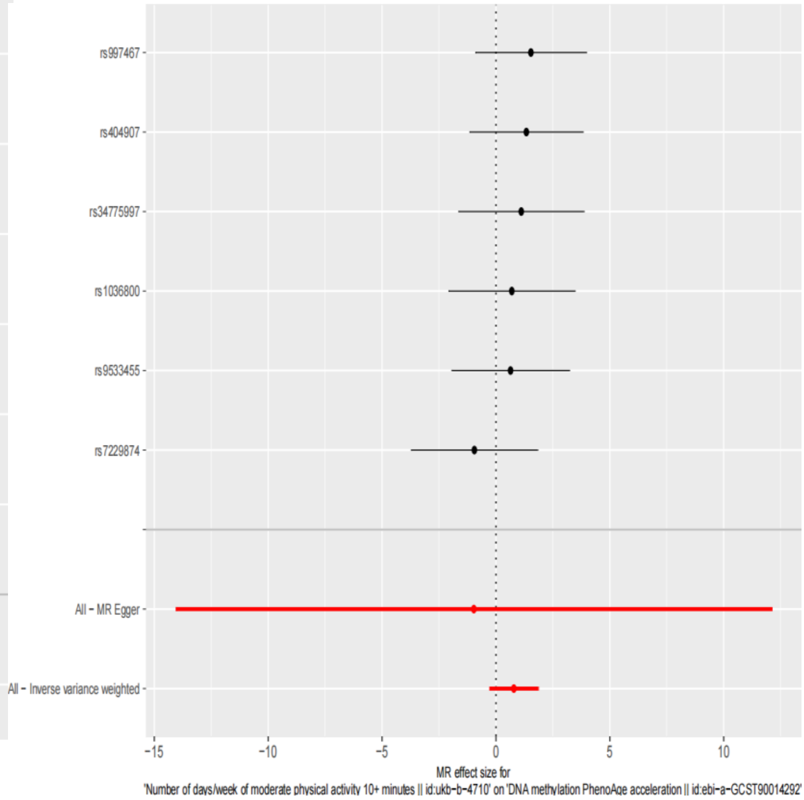
rs997467
rs404907
rs34775997
rs1036800
rs9533455
rs7229874
All − MR Egger
All − Inverse variance weighted
MR effect size for
'Number of days/week of moderate physical activity 10+ minutes || id:ukb−b−4710' on 'DNA methylation PhenoAge acceleration || id:ebi−a−GCST90014292'

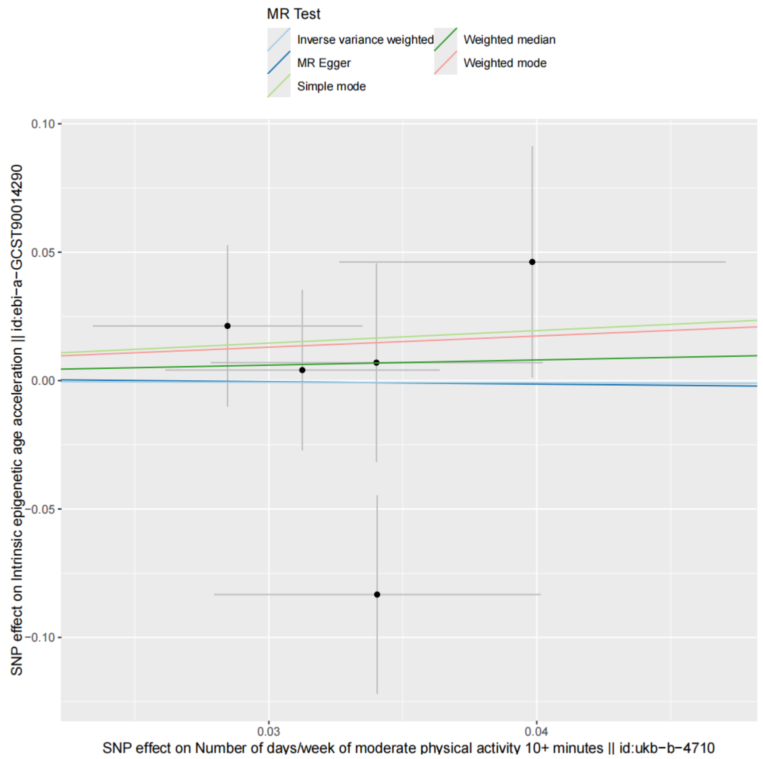
MR Test
Inverse variance weighted
MR Egger
Simple mode
Weighted median
Weighted mode
SNP effect on Intrinsic epigenetic age acceleration || id:ebi-a-GCST90014290
SNP effect on Number of days/week of moderate physical activity 10+ minutes || id:ukb-b-4710

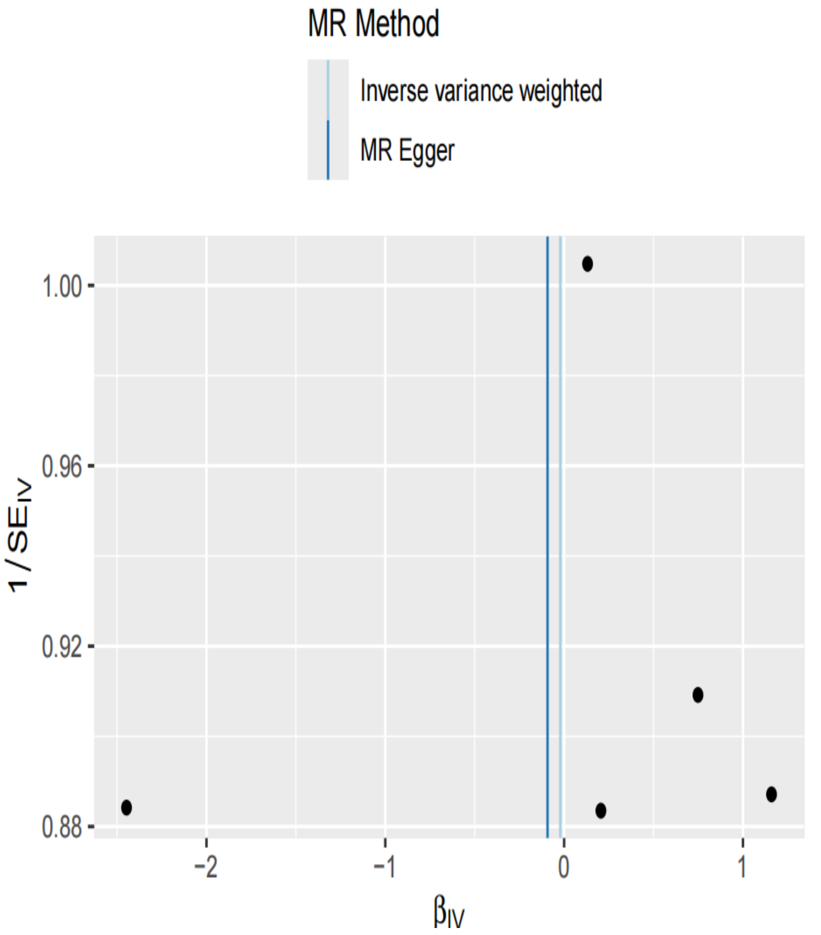
MR Method
Inverse variance weighted
MR Egger
1/SE_IV
β_IV

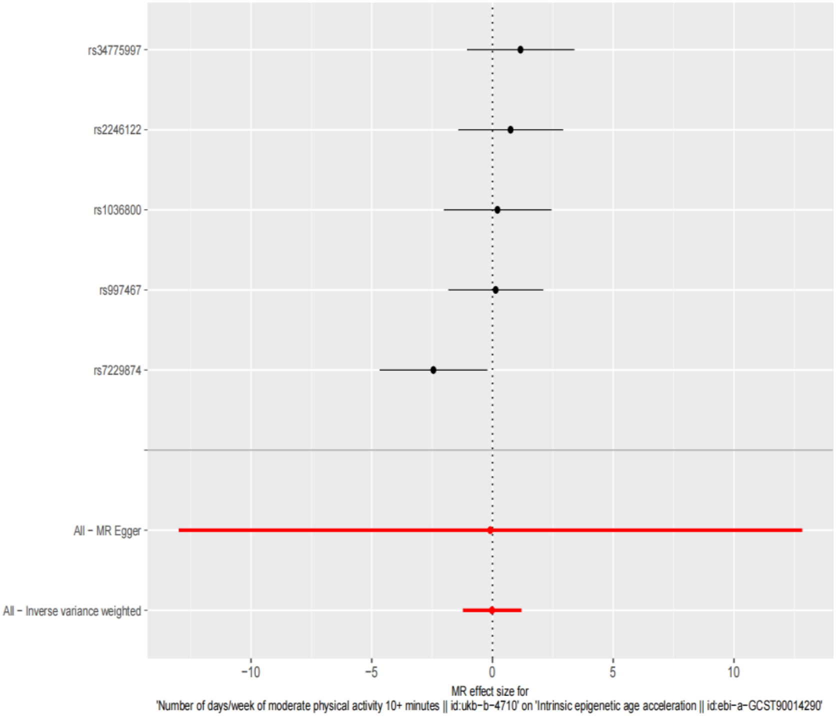
rs34775997
rs2246122
rs1036800
rs997467
rs7229874
All - MR Egger
All - Inverse variance weighted
MR effect size for
'Number of days/week of moderate physical activity 10+ minutes || id:ukb-b-4710' on 'Intrinsic epigenetic age acceleration || id:ebi-a-GCST90014290'

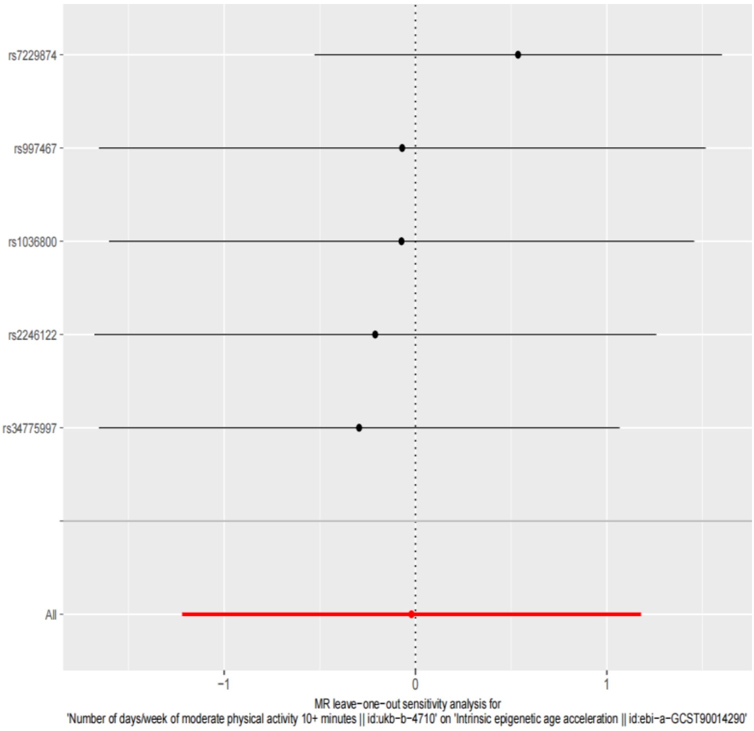
rs7229874
rs997467
rs1036800
rs2246122
rs34775997
All
MR leave-one-out sensitivity analysis for
'Number of days/week of moderate physical activity 10+ minutes || id:ukb-b-4710' on 'Intrinsic epigenetic age acceleration || id:ebi-a-GCST90014290'

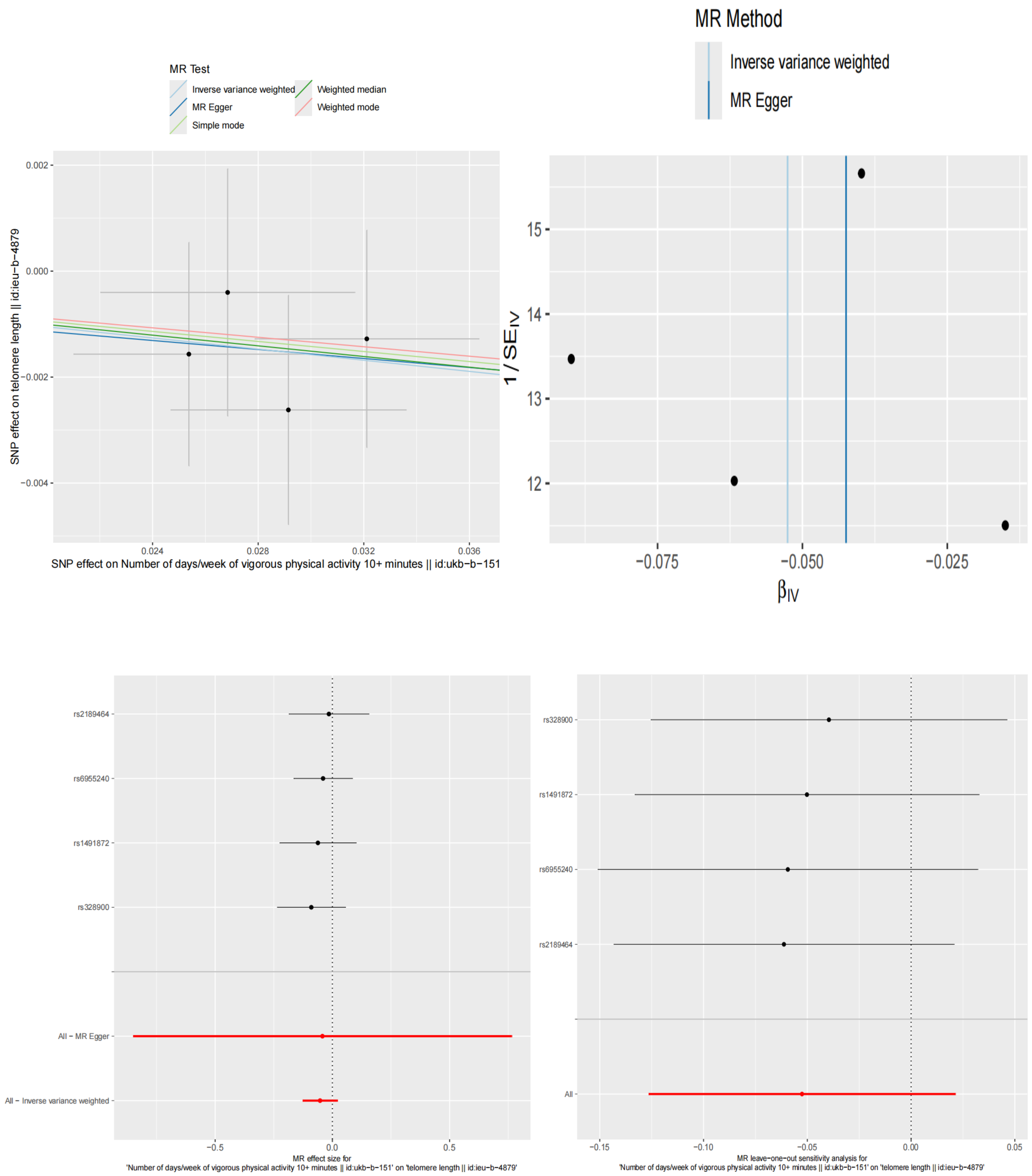

MR Test
Inverse variance weighted
MR Egger
Simple mode
Weighted median
Weighted mode
SNP effect on telomere length || id:ieu−b−4879
SNP effect on Number of days/week of vigorous physical activity 10+ minutes || id:ukb−b−151
MR Method
Inverse variance weighted
MR Egger
1 / SE$_{IV}$
β$_{IV}$
rs2189464
rs6955240
rs1491872
rs328900
All − MR Egger
All − Inverse variance weighted
MR effect size for
'Number of days/week of vigorous physical activity 10+ minutes || id:ukb−b−151' on 'telomere length || id:ieu−b−4879'
All
MR leave−one−out sensitivity analysis for
'Number of days/week of vigorous physical activity 10+ minutes || id:ukb−b−151' on 'telomere length || id:ieu−b−4879'

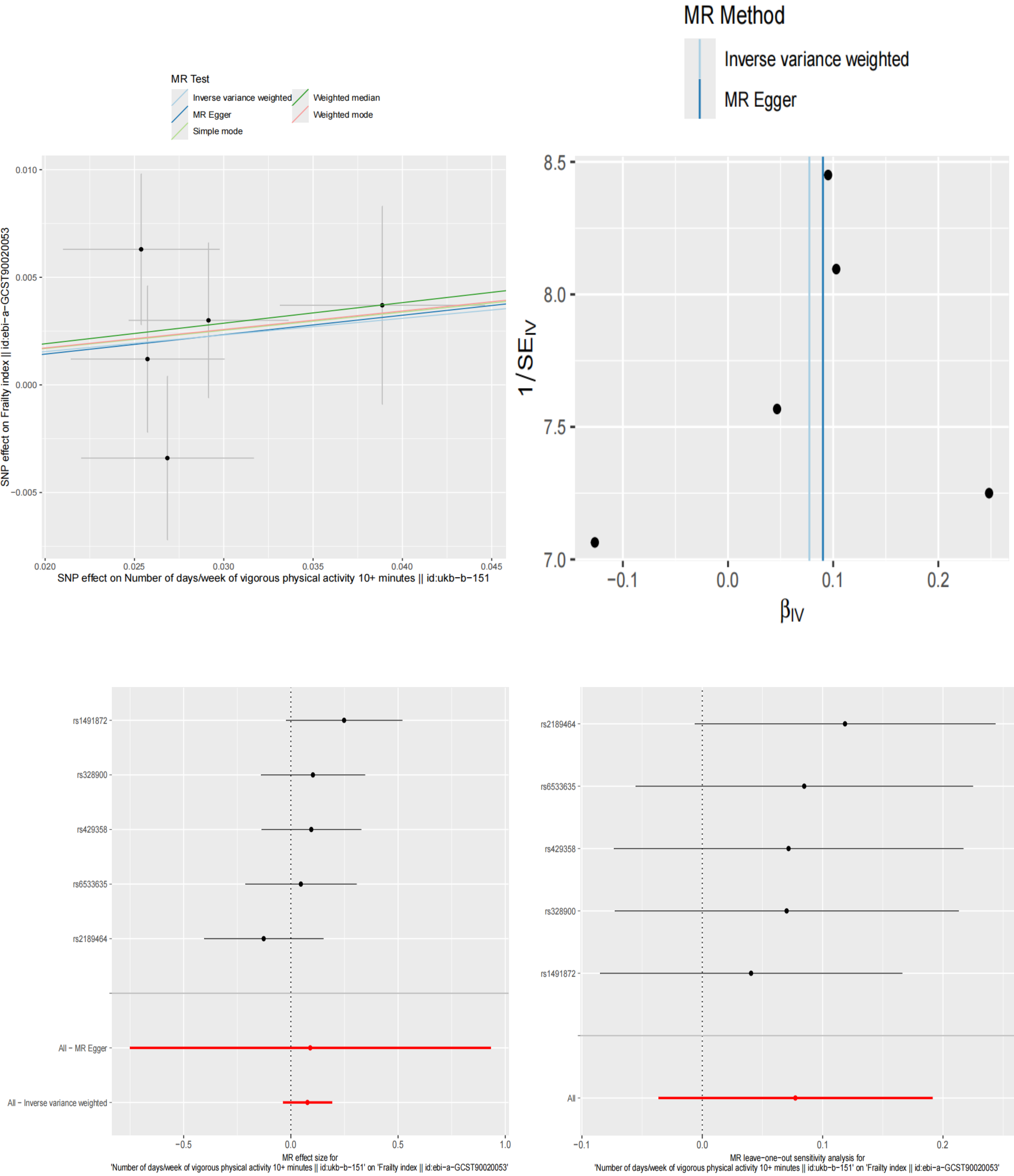
MR Test
Inverse variance weighted
MR Egger
Simple mode
Weighted median
Weighted mode
SNP effect on Frailty index || id:ebi−a−GCST90020053
SNP effect on Number of days/week of vigorous physical activity 10+ minutes || id:ukb−b−151
MR Method
Inverse variance weighted
MR Egger
1/SE$_{IV}$
β$_{IV}$
rs1491872
rs328900
rs429358
rs6533635
rs2189464
All − MR Egger
All − Inverse variance weighted
MR effect size for
'Number of days/week of vigorous physical activity 10+ minutes || id:ukb−b−151' on 'Frailty index || id:ebi−a−GCST90020053'
rs2189464
rs6533635
rs429358
rs328900
rs1491872
All
MR leave−one−out sensitivity analysis for
'Number of days/week of vigorous physical activity 10+ minutes || id:ukb−b−151' on 'Frailty index || id:ebi−a−GCST90020053'

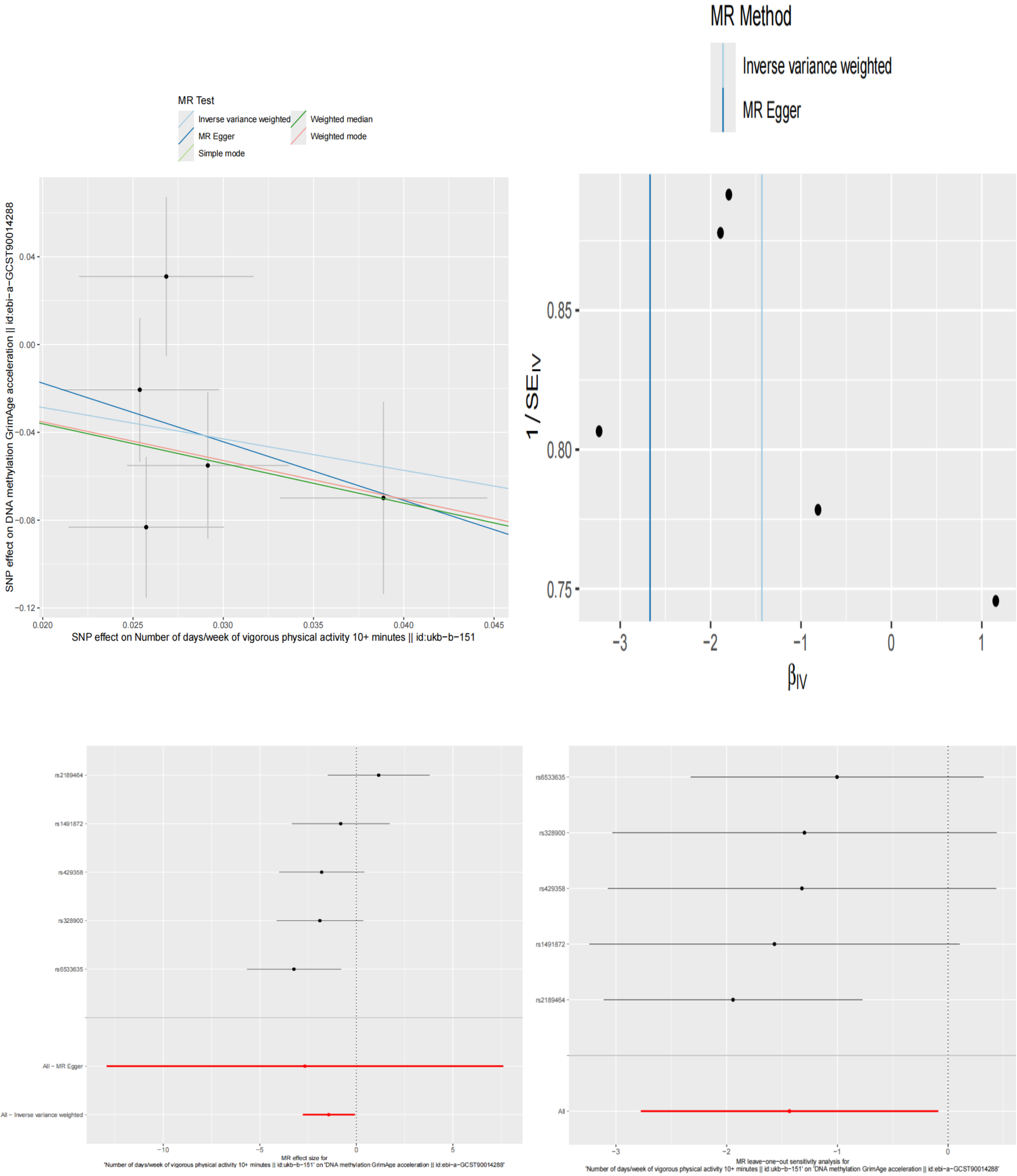

MR Test
Inverse variance weighted
MR Egger
Simple mode
Weighted median
Weighted mode
SNP effect on DNA methylation GrimAge acceleration || id:ebi-a-GCST90014288
SNP effect on Number of days/week of vigorous physical activity 10+ minutes || id:ukb-b-151
MR Method
Inverse variance weighted
MR Egger
1 / SEIV
βIV
rs2189464
rs1491872
rs429358
rs328900
rs6533635
All - MR Egger
All - Inverse variance weighted
MR effect size for
'Number of days/week of vigorous physical activity 10+ minutes || id:ukb-b-151' on 'DNA methylation GrimAge acceleration || id:ebi-a-GCST90014288'
All
MR leave-one-out sensitivity analysis for
'Number of days/week of vigorous physical activity 10+ minutes || id:ukb-b-151' on 'DNA methylation GrimAge acceleration || id:ebi-a-GCST90014288'

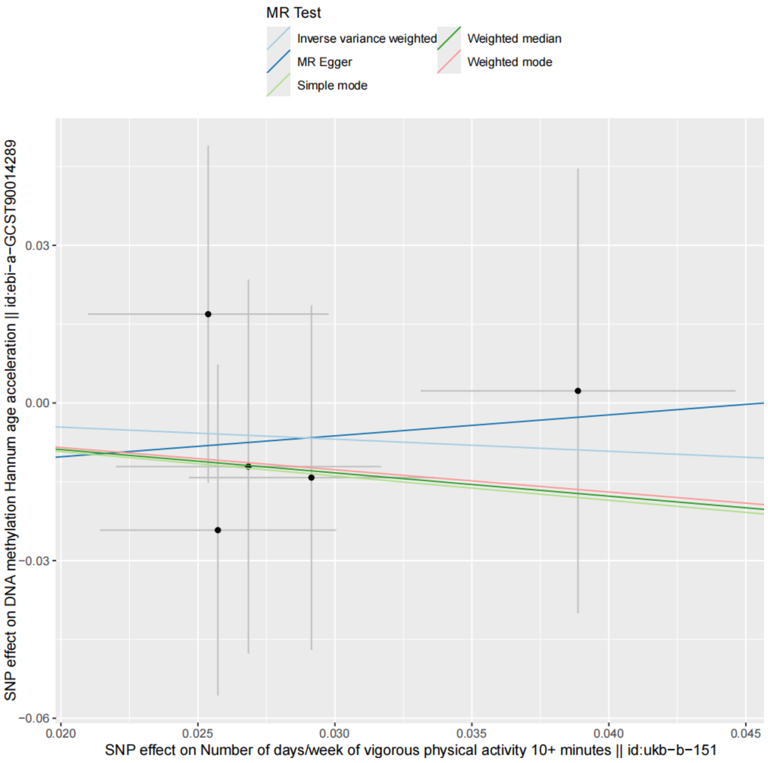

MR Test
Inverse variance weighted
MR Egger
Simple mode
Weighted median
Weighted mode
SNP effect on DNA methylation Hannum age acceleration || id:ebi−a−GCST90014289
0.03
0.00
−0.03
−0.06
0.020
0.025
0.030
0.035
0.040
0.045
SNP effect on Number of days/week of vigorous physical activity 10+ minutes || id:ukb−b−151

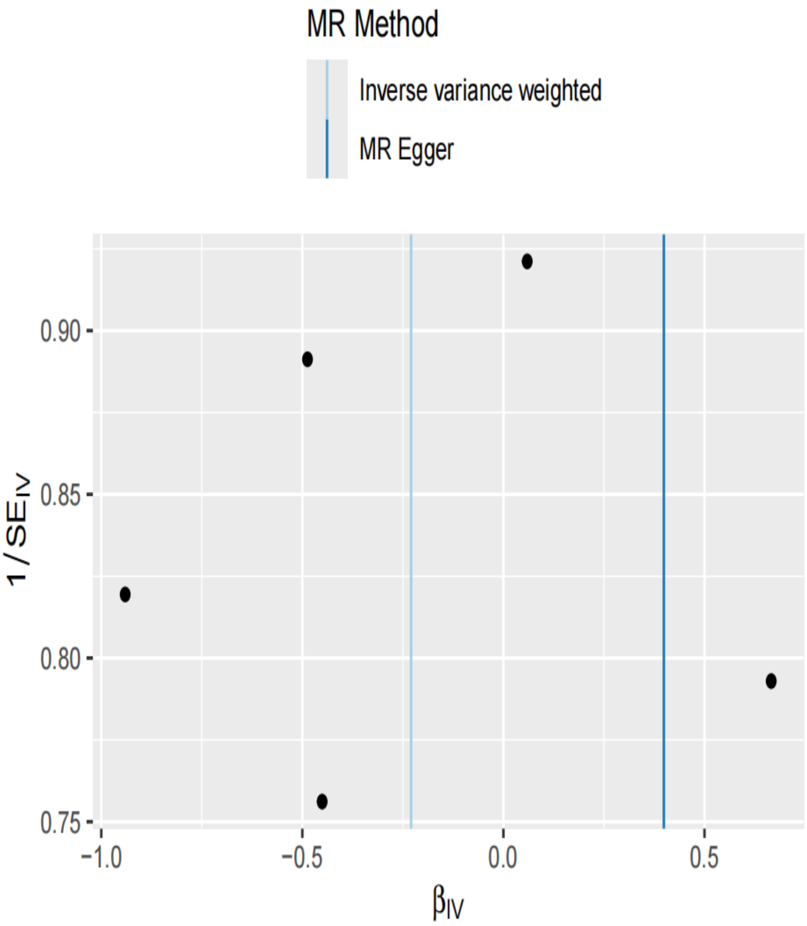

MR Method
Inverse variance weighted
MR Egger
1/SE_IV
0.90
0.85
0.80
0.75
−1.0
−0.5
0.0
0.5
β_IV

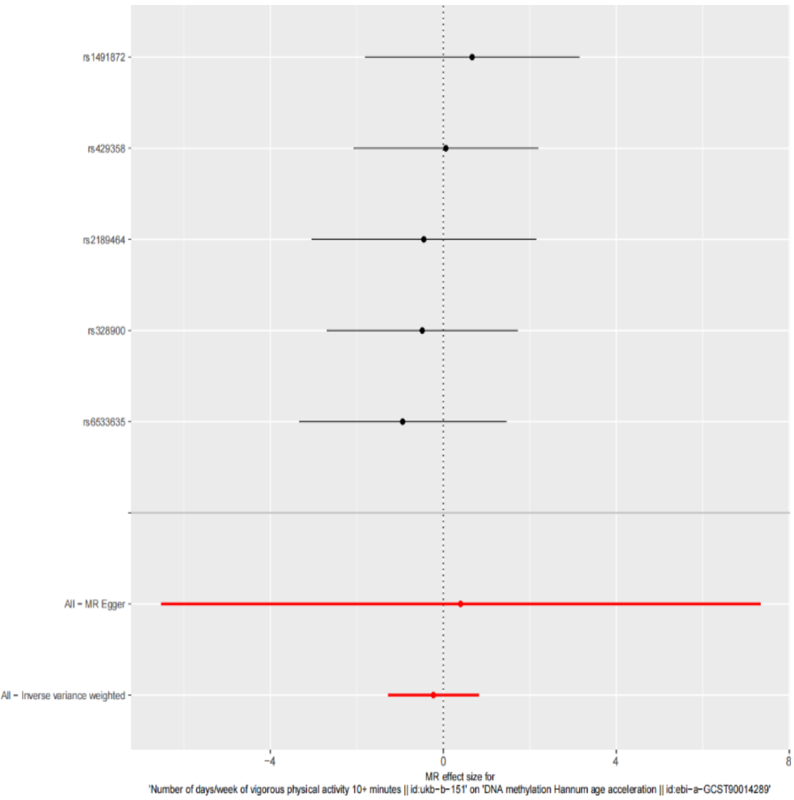

rs1491872
rs429358
rs2189464
rs328900
rs6533635
All − MR Egger
All − Inverse variance weighted
-4
0
4
8
MR effect size for
'Number of days/week of vigorous physical activity 10+ minutes || id:ukb−b−151' on 'DNA methylation Hannum age acceleration || id:ebi−a−GCST90014289'

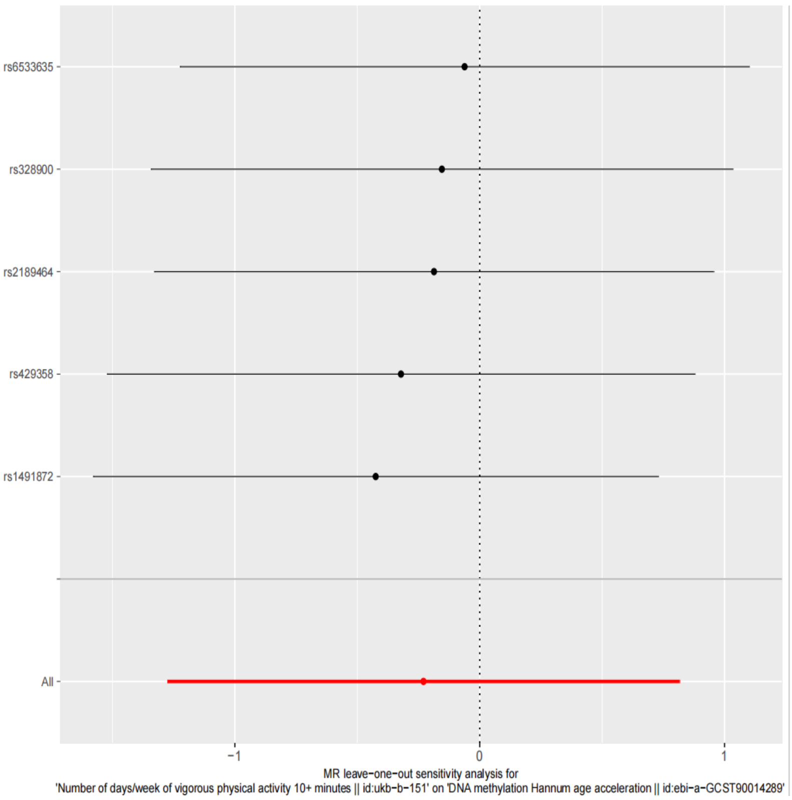

rs6533635
rs328900
rs2189464
rs429358
rs1491872
All
−1
0
1
MR leave−one−out sensitivity analysis for
'Number of days/week of vigorous physical activity 10+ minutes || id:ukb−b−151' on 'DNA methylation Hannum age acceleration || id:ebi−a−GCST90014289'

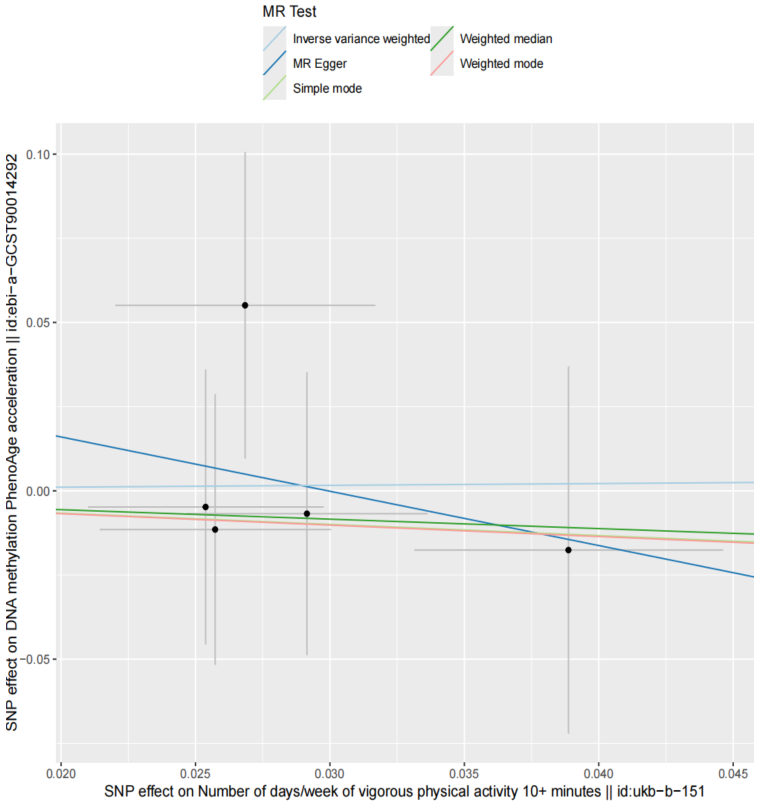
MR Test
Inverse variance weighted
MR Egger
Simple mode
Weighted median
Weighted mode
SNP effect on DNA methylation PhenoAge acceleration || id:ebi-a-GCST90014292
0.10
0.05
0.00
−0.05
0.020
0.025
0.030
0.035
0.040
0.045
SNP effect on Number of days/week of vigorous physical activity 10+ minutes || id:ukb-b-151

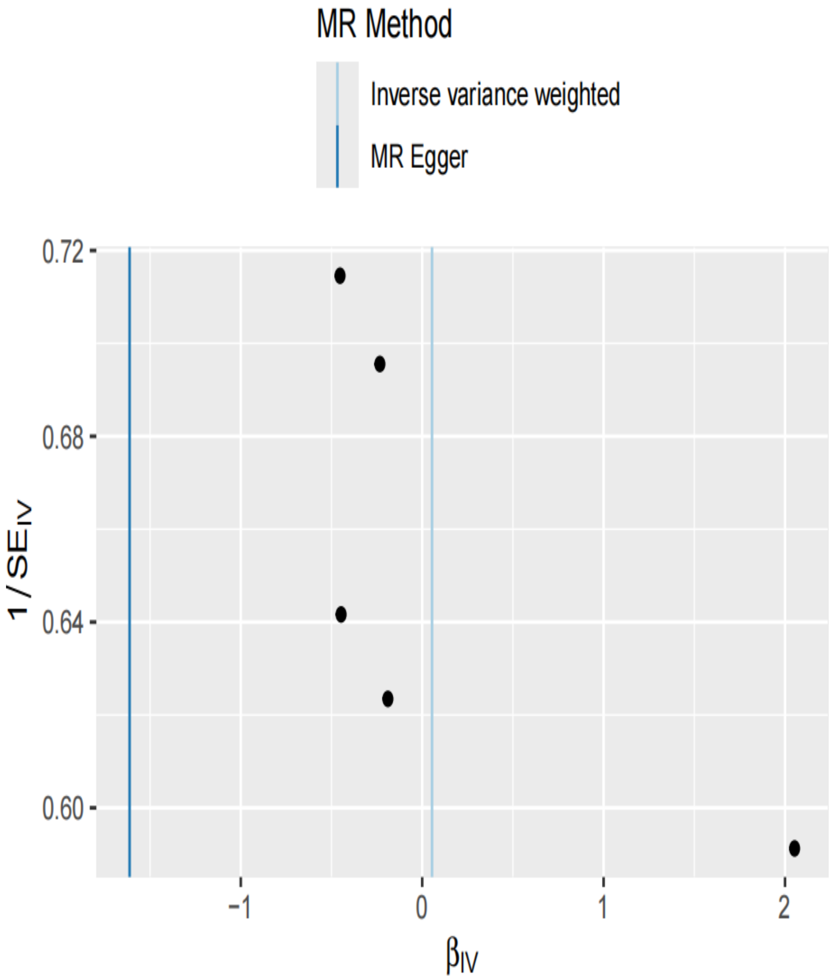
MR Method
Inverse variance weighted
MR Egger
0.72
0.68
0.64
0.60
1 / SEIV
-1
0
1
2
βIV

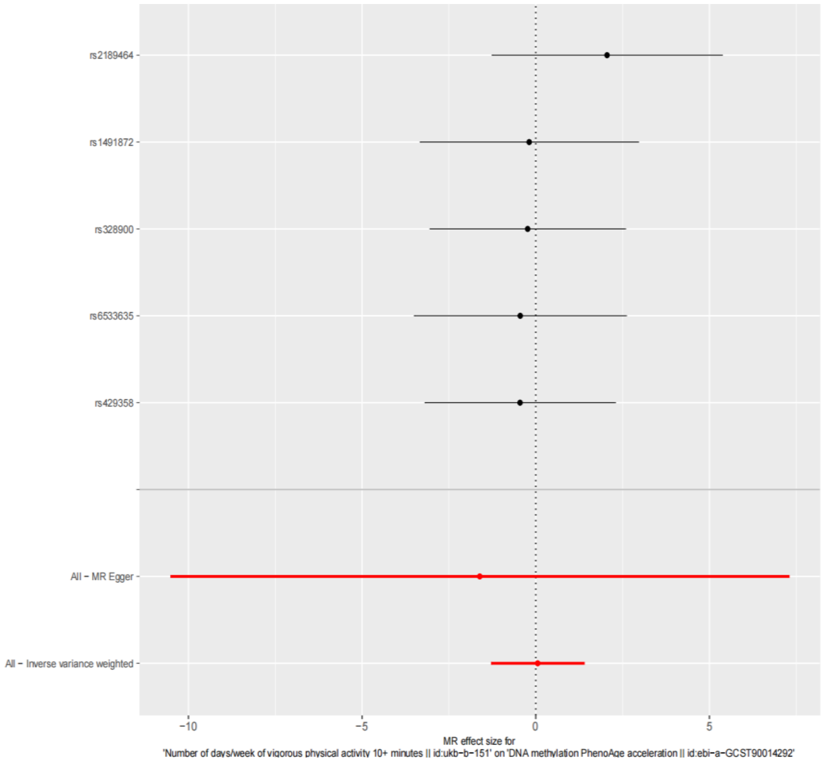
rs2189464
rs1491872
rs328900
rs6533635
rs429358
All − MR Egger
All − Inverse variance weighted
−10
−5
0
5
MR effect size for
'Number of days/week of vigorous physical activity 10+ minutes || id:ukb−b−151' on 'DNA methylation PhenoAge acceleration || id:ebi−a−GCST90014292'

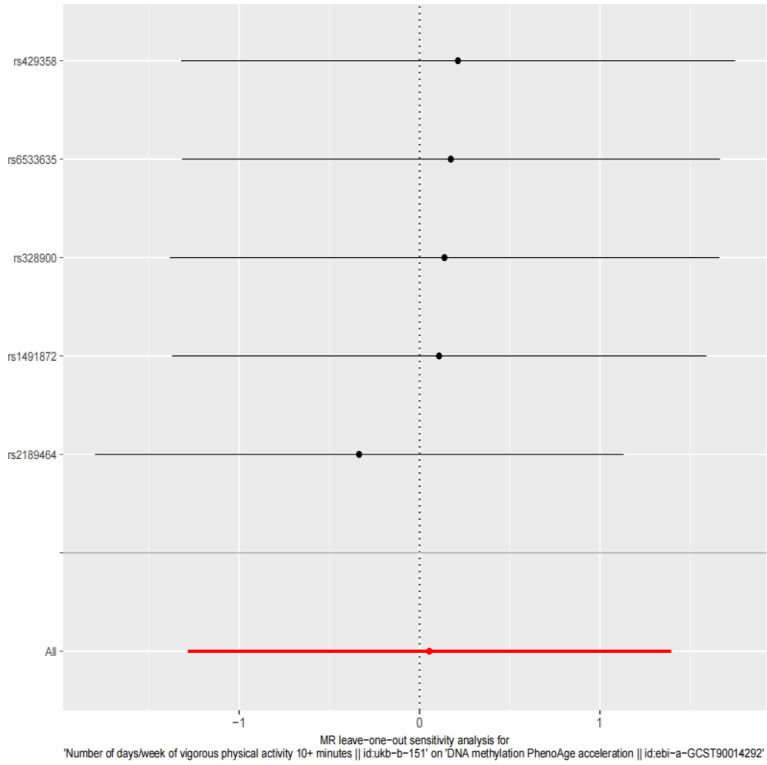
rs429358
rs6533635
rs328900
rs1491872
rs2189464
All
−1
0
1
MR leave−one−out sensitivity analysis for
'Number of days/week of vigorous physical activity 10+ minutes || id:ukb−b−151' on 'DNA methylation PhenoAge acceleration || id:ebi−a−GCST90014292'

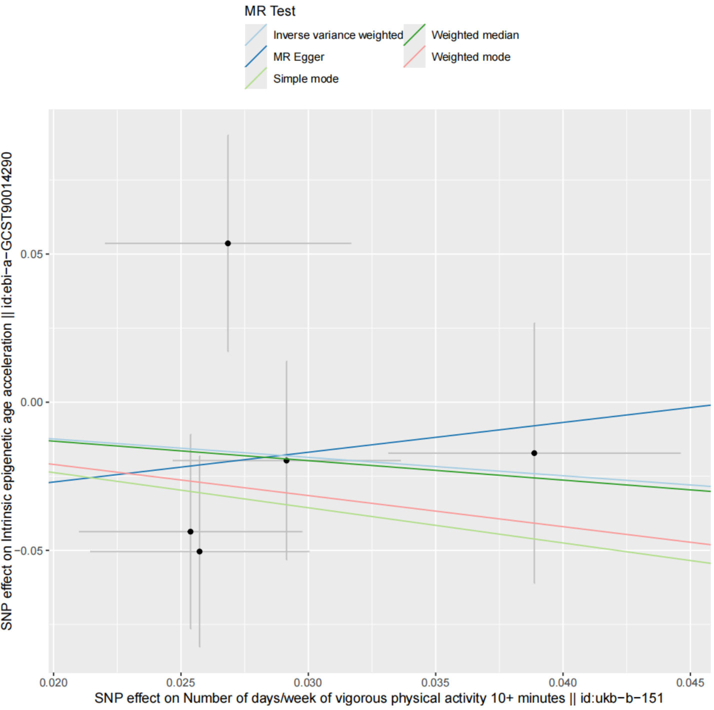
MR Test
Inverse variance weighted
MR Egger
Simple mode
Weighted median
Weighted mode
SNP effect on Intrinsic epigenetic age acceleration || id:ebi-a-GCST90014290
0.05
0.00
-0.05
0.020
0.025
0.030
0.035
0.040
0.045
SNP effect on Number of days/week of vigorous physical activity 10+ minutes || id:ukb-b-151

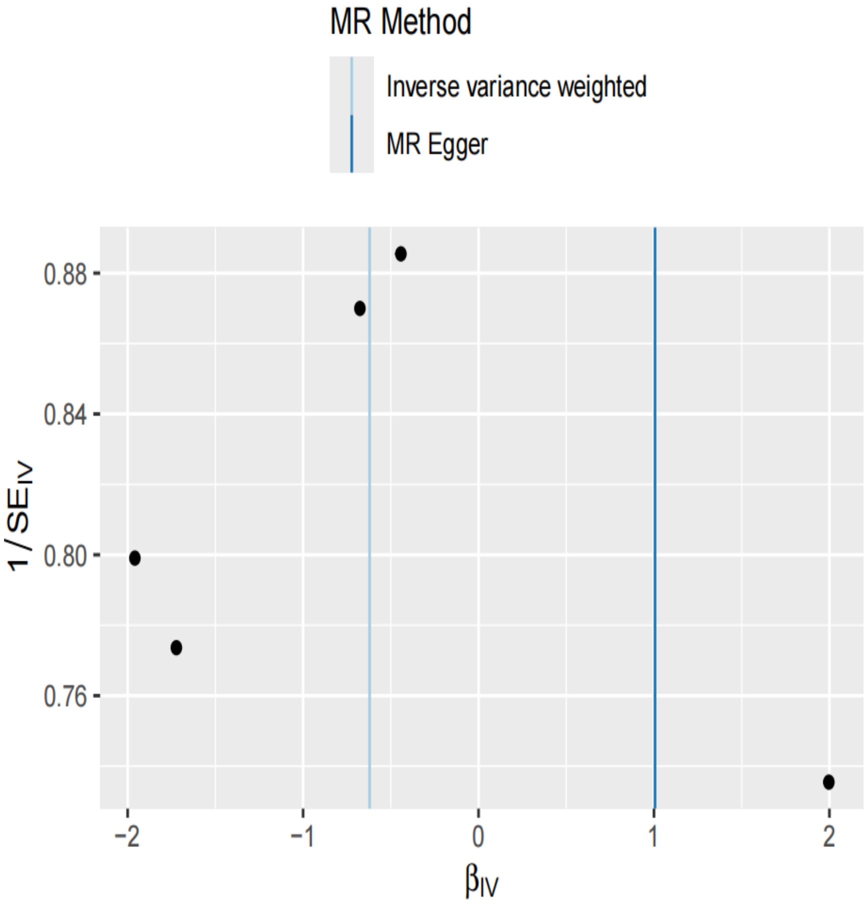
MR Method
Inverse variance weighted
MR Egger
1 / SEIV
0.88
0.84
0.80
0.76
-2
-1
0
1
2
βIV

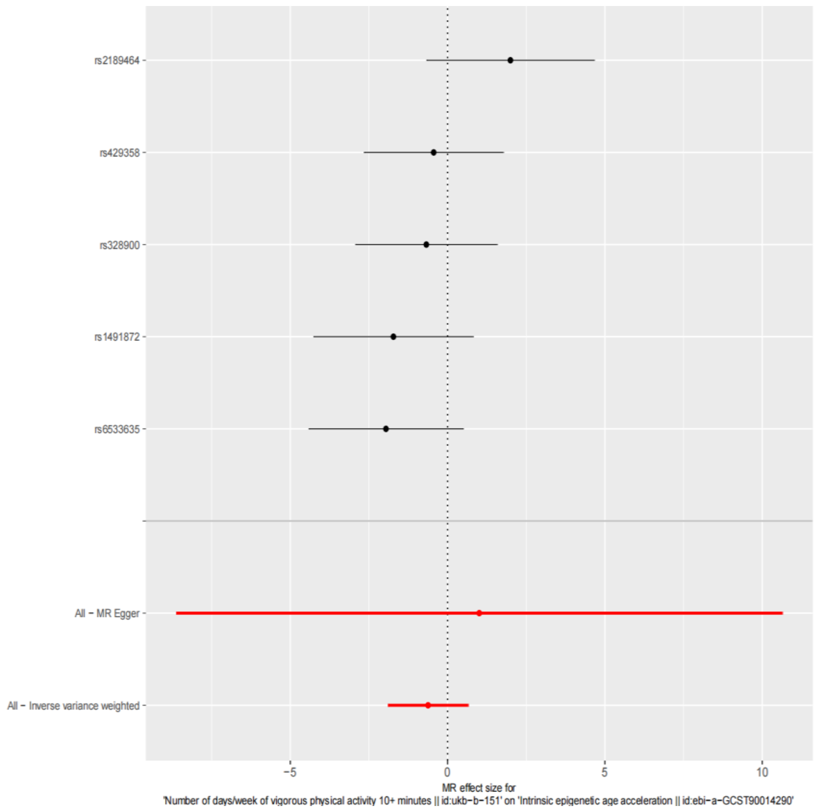
rs2189464
rs429358
rs328900
rs1491872
rs6533635
All - MR Egger
All - Inverse variance weighted
-5
0
5
10
MR effect size for
'Number of days/week of vigorous physical activity 10+ minutes || id:ukb-b-151' on 'Intrinsic epigenetic age acceleration || id:ebi-a-GCST90014290'

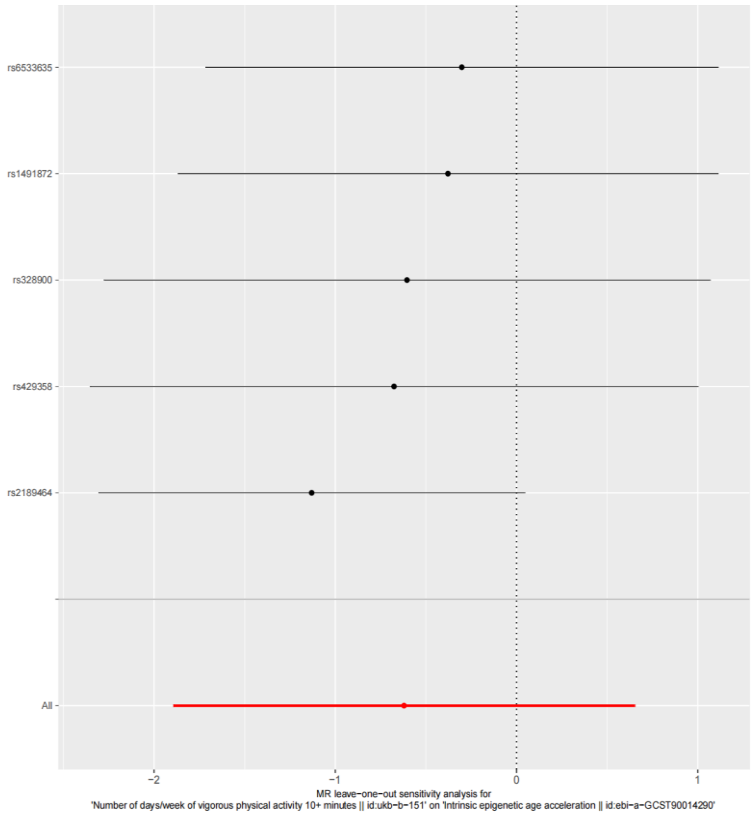
rs6533635
rs1491872
rs328900
rs429358
rs2189464
All
-2
-1
0
1
MR leave-one-out sensitivity analysis for
'Number of days/week of vigorous physical activity 10+ minutes || id:ukb-b-151' on 'Intrinsic epigenetic age acceleration || id:ebi-a-GCST90014290'
